# Bacteriocyte Reprogramming to Cope With Nutritional Stress in a Phloem Sap Feeding Hemipteran, the Pea Aphid *Acyrthosiphon pisum*

**DOI:** 10.3389/fphys.2018.01498

**Published:** 2018-10-25

**Authors:** Stefano Colella, Nicolas Parisot, Pierre Simonet, Karen Gaget, Gabrielle Duport, Patrice Baa-Puyoulet, Yvan Rahbé, Hubert Charles, Gérard Febvay, Patrick Callaerts, Federica Calevro

**Affiliations:** ^1^Univ Lyon, INSA-Lyon, INRA, BF2I, UMR0203, F-69621, Villeurbanne, France; ^2^Laboratory of Behavioral and Developmental Genetics, Department of Human Genetics, KU Leuven, Leuven, Belgium

**Keywords:** pea aphid, symbiosis, bacteriocyte, amino acid stress, phenylalanine and tyrosine pathway, transcriptome profiling

## Abstract

Nutritional symbioses play a central role in the ability of insects to thrive on unbalanced diets and in ensuring their evolutionary success. A genomic model for nutritional symbiosis comprises the hemipteran *Acyrthosiphon pisum*, and the gamma-3-proteobacterium, *Buchnera aphidicola*, with genomes encoding highly integrated metabolic pathways. *A. pisum* feeds exclusively on plant phloem sap, a nutritionally unbalanced diet highly variable in composition, thus raising the question of how this symbiotic system responds to nutritional stress. We addressed this by combining transcriptomic, phenotypic and life history trait analyses to determine the organismal impact of deprivation of tyrosine and phenylalanine. These two aromatic amino acids are essential for aphid development, are synthesized in a metabolic pathway for which the aphid host and the endosymbiont are interdependent, and their concentration can be highly variable in plant phloem sap. We found that this nutritional challenge does not have major phenotypic effects on the pea aphid, except for a limited weight reduction and a 2-day delay in onset of nymph laying. Transcriptomic analyses through aphid development showed a prominent response in bacteriocytes (the core symbiotic tissue which houses the symbionts), but not in gut, thus highlighting the role of bacteriocytes as major modulators of this homeostasis. This response does not involve a direct regulation of tyrosine and phenylalanine biosynthetic pathway and transporter genes. Instead, we observed an extensive transcriptional reprogramming of the bacteriocyte with a rapid down-regulation of genes encoding sugar transporters and genes required for sugar metabolism. Consistently, we observed continued overexpression of the *A. pisum* homolog of RRAD, a small GTPase implicated in repressing aerobic glycolysis. In addition, we found increased transcription of genes involved in proliferation, cell size control and signaling. We experimentally confirmed the significance of these gene expression changes detecting an increase in bacteriocyte number and cell size *in vivo* under tyrosine and phenylalanine depletion. Our results support a central role of bacteriocytes in the aphid response to amino acid deprivation: their transcriptional and cellular responses fine-tune host physiology providing the host insect with an effective way to cope with the challenges posed by the variability in composition of phloem sap.

## Introduction

Hemiptera are a major group of insects occupying a diverse range of ecological niches. Phloem-feeding hemipterans are among the most destructive insect pests of agriculture and forest crops due to their wide host range, rapid reproduction and ability to vector numerous phytopathogens (Girousse et al., 2005; Brault et al., 2010; Perilla-Henao and Casteel, 2016). These insects are the only group of animals using phloem sap, a nutritionally unbalanced diet rich in carbohydrates (e.g., sucrose) but poor in essential amino acids (Hayashi and Chino, 1986; Douglas, 1993; Sandström and Pettersson, 1994; Karley et al., 2002; Dinant et al., 2010), as their predominant or sole food source (Douglas, 2006). All phloem-feeding hemipterans are capable of utilizing phloem sap thanks to their intimate association with symbiotic bacteria which furnish them with (or cooperate with them for the production of) vitamins and/or amino acids otherwise lacking in this food source (Akman Gündüz and Douglas, 2009). In addition to being nutritionally unbalanced, plant phloem sap shows considerable spatial and temporal variability in composition dependent on, e.g., plant species, environment, age of the plant and position of the insect on the plant, all making this a challenging food source for these insects (Sharkey and Pate, 1976; Smith and Milburn, 1980; Hayashi and Chino, 1990; Winter et al., 1992; Corbesier et al., 2001; Douglas, 2003, 2006; Gholami, 2004). Despite their ecological and agronomical importance, how hemipterans and their symbiotic bacteria cope with changes in phloem sap composition and the nature of the molecular responses to these changes are poorly understood.

The pea aphid *Acyrthosiphon pisum* is an excellent hemipteran model to study the interactions between animals and their resident microorganisms. First, its parthenogenetic reproduction allows studying large numbers of individuals of a single genotype. Furthermore, the pea aphid primary symbiont, the gamma-3-proteobacterium, *Buchnera aphidicola*, which has coevolved with its host for over 150 million years, is one of the best characterized symbiotic bacteria (Moran et al., 1993; Moran and Baumann, 1994; Von Dohlen and Moran, 2000; Tagu et al., 2010).

The genomes of the pea aphid and its primary symbiont have both been sequenced and their analysis revealed that these two obligate mutualists are fully interdependent for the biosynthesis of amino acids with the two genomes forming a highly integrated metabolic network (Shigenobu et al., 2000; International Aphid Genomics Consortium, 2010). The amino acid biosynthetic pathways illustrate the metabolic interdependence of host and endosymbionts. Both partners participate in the biosynthesis of the 10 amino acids: arginine, cysteine, glycine, isoleucine, leucine, lysine, methionine, phenylalanine, threonine, and valine. Histidine and tryptophan are synthesized exclusively by *Buchnera*, which in turn is auxotrophic for the biosynthesis of alanine, asparagine, aspartate, glutamine, glutamate, proline, serine, and tyrosine (Wilson et al., 2010; Hansen and Moran, 2011; Poliakov et al., 2011; Wilson, 2011; Wilson and Duncan, 2015).

The tyrosine (Tyr) and phenylalanine (Phe) biosynthetic pathway is one of the most integrated, consisting of a network of genes encoded by the genomes of host and primary symbiont. *B. aphidicola* produces all the necessary precursors to synthesize Phe and Tyr, but has lost the terminal biosynthetic enzymes of the pathway and fully relies on the host insect for their biosynthesis (International Aphid Genomics Consortium, 2010; Wilson et al., 2010). The exchange of precursors and terminal products between the host and the symbiont is often referred to as the aromatic shuttle (Rahbé et al., 2002), where (i) the endosymbiont provides the host with phenylpyruvate the direct precursor of Phe, (ii) the aphid synthesizes Phe from this precursor, and subsequently Tyr from Phe, and (iii) the aphid supplies both its own cells and *B. aphidicola* with Phe and Tyr (Wilson et al., 2010; Simonet et al., 2016b). Several studies have demonstrated the importance of Tyr and Phe for aphid performance, and embryonic development (Douglas, 1996; Wilkinson and Ishikawa, 1999, 2000; Bermingham and Wilkinson, 2010). The Tyr/Phe pathway is activated during pea aphid parthenogenetic development, with several enzyme-encoding genes being up-regulated in the late phases of embryonic development and at the beginning of nymphal development (Rabatel et al., 2013). Recently, Simonet et al. (2016b) confirmed the functional importance of this pathway in aphid development through the disruption of phenylalanine hydroxylase (ApPAH, EC:1.14.16.1). This enzyme catalyzes the conversion of Phe to Tyr, and is only encoded by the host genome. *ApPAH* gene inactivation shortened the adult aphid lifespan and considerably affected fecundity by diminishing the number of produced nymphs and impairing embryonic development, with severe morphological defects in the progeny.

In apparent contrast to the fact that these aromatic amino acids are important for aphid development and growth, Tyr and Phe have been reported to be in low abundance in plant sap (from trace amounts to 0.5–4%) (Hayashi and Chino, 1990; Karley et al., 2002; Douglas, 2006). This thus raises the important question of how this symbiotic system copes with fluctuations in the concentrations of these two amino acids.

Organisms facing environmental constraints often display extensive transcriptional plasticity (modulation of gene expression). However, similar to other symbiotic bacteria, the *B. aphidicola* genome has undergone drastic size reduction (Gil et al., 2002) and most of the classical bacterial gene expression regulatory networks are missing [reviewed in Brinza et al. (2009)]. Several studies have indicated the lack of a strong and specific transcriptional response of this bacterium following a stress applied to the aphid host (Moran et al., 2005; Wilson et al., 2006). Furthermore, Reymond et al. (2006) demonstrated that the transcriptional response of *B. aphidicola* from aphids reared on a Tyr/Phe depleted diet was not specifically oriented toward aphid needs. These results led us to hypothesize that the host regulates the response to nutrient stress.

To test this hypothesis and uncover the aphid response at the molecular level we used a chemically defined artificial diet to characterize the impact of deprivation of Tyr and Phe on *A. pisum* transcriptome and life history traits. The complete control diet (AP3) supports normal development, feeding and reproduction (Febvay et al., 1988). Selective removal of Tyr and Phe (YFØ diet) has the advantage to create a framework to identify the cellular and molecular basis of the response of *A. pisum*/*B. aphidicola* to naturally occurring fluctuations in phloem sap composition. We focused on gut and bacteriocytes because the gut epithelium is known to act as a regulatory hub in insect nutrition (Huang et al., 2015) while bacteriocytes represent the core symbiotic tissue, as they house the symbiotic bacteria (Buchner, 1965; Baumann et al., 1995). Our analysis revealed that the pea aphid does not show major phenotypic differences under this nutritional challenge. Moreover, the gut transcriptome is not significantly altered under nutrient stress. By contrast, the major response to this nutritional constraint occurs at the level of the bacteriocyte cell. Surprisingly, these alterations do not involve a modulation of the tyrosine and phenylalanine biosynthetic pathways to directly compensate for the depletion of these two amino acids from the aphid diet. Instead, we discovered a transcriptional reprogramming of bacteriocyte cells including a reduction in expression of genes required for sugar metabolism, a modulation of the transport function, and increased transcription of genes related to cell growth, proliferation and associated signaling pathways. We experimentally validated these observations *in vivo* detecting a significant increase in bacteriocyte number and size. Our findings are indicative of the transcriptional and phenotypic plasticity of bacteriocyte cells. Given the universal presence of bacteriocytes in hemipterans and other symbiotic insects, and their central role in the interactions of these insects with their symbionts, we anticipate that equivalent mechanisms may be present in other symbiotic systems that live and reproduce on nutritionally unbalanced diets.

## Materials and Methods

### Aphid Rearing and Performance Measures

A long-established parthenogenetic clone (LL01) of *A. pisum* (Harris), devoid of secondary symbionts, was maintained on young broad bean plants (*Vicia faba*, L. cv. Aquadulce), at 21°C, with a 16 h photoperiod. In order to obtain a source of synchronized apterous parthenogenetic aphids, winged adults were left on seedlings, to allow them to produce nymphs, and were removed after 24 h. Synchronized N1 nymphal instars were then transferred to two artificial diets differing only in their amino acid composition: the AP3 and the YFØ diets (see section “Artificial Diets” for details). N1 nymphs (30 for the AP3 and 30 for the YFØ diets, respectively) were left to develop for 30 days and were checked daily for survival and the presence of possible different phenotypic effects. Aphids were weighed at Day 7, the time point of transition to adulthood in aphids reared on plants. Ten aphids per diet were isolated and followed individually for fecundity: the number of newborn nymphs was counted, their size measured with a Leica MZFLIII (Leica, Wetzlar, Germany) microscope using an F-view camera link to the CellF software (Soft Imaging 197 System, Tokyo, Japan) and they were checked for any visible morphological phenotype.

### Artificial Diets

The composition of the artificial diet (AP3) was as originally defined by us combining data on the amino acid composition in both the phloem sap of leguminous plants and aphid hemolymph (Febvay et al., 1988). The YFØ diet is the same as AP3 minus tyrosine (Y) and phenylalanine (F). Following the preparation of new aliquots of the artificial diets, actual amino acid concentrations are determined by HPLC analysis (Febvay et al., 1999). Furthermore, for the AP3 control diet, aphid life history traits, survival and honeydew production are measured prior to use in experiments (see also the section “Results”). On this diet, aphid survival is standardly comparable to that of aphids reared on plants.

### Sampling of Aphid Tissues for RNA Extraction

**FIGURE 1:**
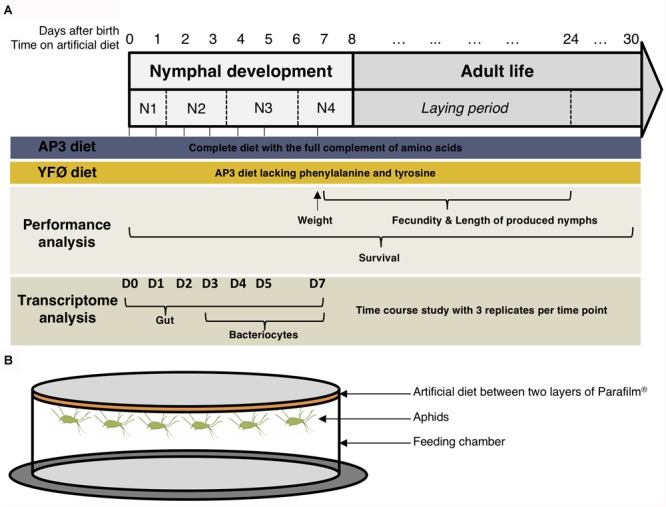
**(A)** Experimental design for the overall assessment of the *Acyrthosiphon pisum* performance and tissue-specific gene expression changes underlying the response to a tyrosine and phenylalanine depleted diet. **(B)** Schematic illustration of the artificial diet feeding chamber.

This study represents the first time-course analysis of *A. pisum* gene expression on a genome-wide scale (see Figure 1 for the experimental design). Aphids were dissected in ice-cold iso-osmotic buffer A (pH 7.5, 0.025 M KCl, 0.01 M MgCl_2_, 0.25 M Sucrose, and 0.035 M Tris–HCl) under 25–40× magnification with an MDG-17 stereomicroscope (Leica) and two tissues were isolated: the gut and the bacteriocytes. All collected nymphs were randomly selected from the synchronized source population. Gut samples were isolated at seven distinct time points following the transfer on the two artificial diets after 12 h (D0), 1 day (D1), 2 days (D2), 3 days (D3), 4 days (D4), 5 days (D5), and 7 days (D7) (Figure 1 and Supplementary Table S1). Three biological replicates were collected per time point (seven) and per diet (two), with each biological replicate consisting of 30 guts. The total number of sample replicates for the gut was 42. Bacteriocyte samples were collected at D3, D4, D5, and D7. Earlier stages did not yield sufficient RNA for subsequent processing and microarrays. Three biological replicates were collected per time point (four) and per diet (two) with each biological replicate containing between 800 and 1000 bacteriocytes. The total number of sample replicates for the bacteriocytes was 24 (Figure 1 and Supplementary Table S1). All dissected tissues were placed in RNAlater^®^ (Thermo Fisher Scientific, Waltham, MA, United States) and stored at -80°C. Further details on sample numbers and size are provided in Supplementary Table S1.

### RNA Extraction

Total RNA was prepared using the RNeasy Mini kit (Qiagen, Hilden, Germany). Total RNA concentration and quality were initially checked using the NanoDrop^®^ ND-1000 Spectrophotometer (Nanodrop Technologies, Wilmington, DE, United States) and samples had to meet the following quality parameters: A260/A280 ≥ 1.8 and A260/A230 ≥ 1.8, in order to be used in the subsequent analysis. The integrity of the RNA samples was checked using the Agilent RNA 6000 Nano Kit and the Agilent 2100 Bioanalyzer (Agilent Technologies, Santa Clara, CA, United States). Only good quality samples responding to the described criteria were used for subsequent analyses.

### Amplification of mRNA and cDNA Synthesis

Microarrays require RNA quantities that exceed what can be isolated from 30 guts or 800–1000 bacteriocytes. Therefore, we applied a commonly used preparatory step for microarrays, i.e., linear amplification of RNA. Linear amplification maintains the relative amounts of existing mRNAs in a cell or tissue without skewing the representation of individual mRNAs in a complex mixture (Schneider et al., 2004). The MessageAmp^TM^ II aRNA Amplification kit (Thermo Fisher Scientific) was used following the manufacturer’s instructions. Based on the total RNA quantification profiles, amplifications of the bacteriocyte samples were conducted on RNA quantities two times greater than for gut samples (0.5 μg) to compensate for the twofold higher prokaryotic rRNA concentrations (16S/23S rRNA peaks) relative to eukaryotic rRNA (18S/28S) in bacteriocytes.

The amplified RNA (aRNA) was used to prepare double stranded cDNA with the Superscript II kit (Thermo Fisher Scientific), as recommended by NimbleGen in the NimbleChipTM Arrays User’s Guide for gene expression analysis. Starting with 5 μg of aRNA, the samples were processed according to the manufacturer’s instructions, including these four steps: (i) initial cDNA synthesis using random primers, (ii) second strand synthesis, (iii) RNase A clean-up, and (iv) cDNA precipitation. For each sample, the integrity of the aRNA and cDNA was checked for possible degradation using the Agilent RNA 6000 Nano Kit on the Agilent 2100 Bioanalyzer. Only good quality samples were retained for the microarray experiments performed by Roche NimbleGen (Madison, WI, United States).

### Microarray Experiments and Data Collection

The “INRA-BF2I_A.pisum_Nimblegen-ACYPI_4x72k_v1” microarray for the pea aphid was developed in collaboration with Roche NimbleGen using the pea aphid genome v1.0 assembly (International Aphid Genomics Consortium, 2010). This NimbleGen 385K 4-plex (4 × 72 000 probes) high-density array can accommodate 4 samples that are hybridized onto a section of the array containing 72 000 60-mers oligonucleotide probes, representing 24 011 pea aphid transcripts (corresponding to 23855 genes) as described in Rabatel et al. (2013). The microarray design can be found in the ArrayExpress database (Accession No. A-MEXP-1999). Labeling (using the NimbleGen One-Color DNA Labeling Kits and Cy3 Random Nonamers), hybridization on the arrays (at 42°C for 16–20 h) and scanning (using MS 200 Microarray Scanner and the MS 200 Data Collection Software) were carried out by Roche NimbleGen, as described in the NimbleGen arrays user’s guide for gene expression arrays. All the transcriptomic data obtained are available in the ArrayExpress database (Accession No. E-MTAB-4456).

### Microarray Data Analysis: Quality Control

A first global data analysis using principal component analysis (PCA) revealed that one of the three gut replicates from aphids reared on the AP3 diet for 3 days (Gut-AP3_D3-1) was aberrant (Supplementary Figure S1). This replicate did not cluster with either gut or bacteriocyte samples, and is most likely an incorrectly isolated tissue or a contaminant. It was therefore removed from the subsequent analyses. In order to keep a symmetric design for analysis purposes, one replicate at the same time point from gut in aphids reared on YFØ diet was also removed (Gut-YFØ_D3-1). Note that this sample did cluster with other gut samples (Supplementary Figure S1). Thus, in the final differential expression analysis for the time-point D3, for gut samples, two replicates were used both on the control (AP3) and Tyr/Phe depleted diet (YFØ) samples. 40 gut samples and 24 bacteriocyte samples (total 64) were used for further analyses.

### Microarray Data Analysis: Differential Expression

Microarray data were normalized, using the RMA method (Irizarry et al., 2003), and then transformed into log_2_ for subsequent analyses. Differentially expressed genes (DEGs) were predicted using one-way between groups ANOVA analyses [Limma package (Ritchie et al., 2015) from the R Bioconductor project v3.2]. Moderated *t*-test *P*-values were adjusted using a false discovery rate [FDR, (Dudoit et al., 2003)] threshold of 0.05. Tissue-enriched genes were detected using the control diet (AP3) samples and only the genes harboring a |log_2_(Fold-Change)| > 2 were kept for further analyses. Pairwise comparisons between the two diets at each time point and for each tissue were also performed to identify the DEGs in response to a Tyr and Phe depleted diet. For this contrast, no additional filtering was performed. Interactive graphs were obtained through Cytoscape v3.5.0 (Shannon et al., 2003). All PCA were performed using the R ADE4 package (Dray and Dufour, 2007).

### Microarray Data Analysis: Functional Annotation Analysis

As the microarray was originally designed on the v1.0 of the pea aphid *A. pisum* genome, we filtered the dataset based on the recently released pea aphid genome v2.0 assembly to exclude both outdated genes and genes with cross-hybridizing probes using PatMaN (Prüfer et al., 2008) with a threshold of three mismatches. Using this filter, 13 668 genes were excluded among which 13 646 (99.8%) that were unannotated. Only the remaining genes (10 343) were taken into account for the functional annotation analysis. The Gene Ontology (GO) analysis was first performed using the pea aphid annotation v2.1b. In order to have more detailed results and given the more extensive functional annotation of genes in *Drosophila melanogaster*, we decided to perform a second GO analysis using the corresponding *D. melanogaster* genes for each differentially expressed *A. pisum* gene. For simplicity’s sake, we refer to these genes as “putative homologs” throughout the text. To perform this analysis, we retrieved putative *D. melanogaster* homologs for each differentially expressed *A. pisum* gene using BLASTP (Altschul et al., 1990) and we carried out a GO analysis based on the annotations collected in FlyBase version FB2017_01 (Gramates et al., 2017). Enrichment analysis of the DEG sets was performed using BINGO v3.0.3 (Maere et al., 2005) with an FDR threshold of 0.01. Lists of enriched GO terms were further refined using REVIGO (Supek et al., 2011) with a similarity threshold of 0.5 and the *D. melanogaster* database.

### Microarray Data Validation

To validate the transcriptional differences identified with the microarray analyses, we conducted quantitative reverse transcription-PCR (qRT-PCR) experiments for a selection of genes (seven) corresponding to different functional classes and spanning a broad range of expression differences as determined with the microarrays. Three genes were differentially expressed in the gut samples (*ACYPI000653, ACYPI001701*, and *ACYPI010105*), three genes were differentially expressed in the bacteriocytes (*ACYPI003338, ACYPI004647*, and *ACYPI006800*), and one gene was differentially expressed in both tissues (*ACYPI001281*). Expression of these seven genes was assessed for all the time points used in the microarray experiment (a total number of 35 sample replicates for the guts and 16 sample replicates for the bacteriocytes). Primers to target transcripts (Supplementary Table S2) were designed with the Oligo6 software (Rychlik, 2007). For data normalization, two genes were tested in the different tissues and time-points analyzed: *rpl7* (*ACYPI010200*) and *rpl32* (*ACYPI000074*). Real-time RT-PCR data were analyzed using the BestKeeper© software tool (Pfaffl et al., 2004) and the *rpl7* gene was retained as the best candidate for data normalization.

Internal standard curves were generated for each gene using serial dilutions (from 2000 to 0.002 fg/μl) of purified PCR products amplified from a pool of cDNA. The PCR reaction to prepare the control sample for the standard curve was carried out starting from 1 μl of reverse transcription product using UptiTherm DNA Polymerase (Interchim, Montluçon, France), according to the manufacturer’s instructions.

qRT-PCR analyses were done following essentially the same strategy described in Dallas et al. (2005). aRNA samples were treated with DNase I (Promega, Madison, WI, United States) and reverse-transcribed in cDNA using the SuperScript^TM^ III First-Strand Synthesis System for RT-PCR (Thermo Fisher Scientific), with random primers and oligodT, according to the manufacturer’s instructions. RT-PCR was performed with a LightCycler 480 Instrument (Roche Diagnostics, Basel, Switzerland) using either 2.5 μl of cDNA (at around 1.5 μg/μl), diluted at 1/5, or water (for negative control reactions) in a total PCR reaction final volume of 10 μl. Quantitative RT-PCR data were analyzed using the REST software^1^ (Pfaffl et al., 2002).

The correlation analysis between microarray and qRT-PCR data was done with (i) a regression analysis using a linear model (*R*-squared of 0.8299; *F* = 210.8; d.f. = 42; *P* < 2.2 × 10–16), and (ii) a Pearson’s coefficient of determination.

### Counting and Size Determination of Aphid Bacteriocytes

For cell counting, bacteriocytes were surgically isolated from the abdomen of each individual aphid and counted at 25–40× magnification with an MDG-17 stereomicroscope (Leica). For each time point and each artificial diet, a total number of 10 aphids were analyzed. To determine their size, bacteriocytes were collected with a micropipette and mounted on glass slides. Double spacers made from microscope coverslips, with a thickness of 170 μm each, were used to mount bacteriocytes following the procedure we recently developed (Simonet et al., 2016a; 2018). The total space of 340 μm between microscope slides and the coverslip covering the preparation exceeds the maximal diameter of bacteriocytes (ranging from 40 to 120 μm) thereby preventing any physical damage or deformation of bacteriocytes. Bacteriocyte images were acquired and volumes were calculated as previously described (Simonet et al., 2016a). At least seven aphids were analyzed for each time point and each artificial diet. A total of 700 bacteriocytes were analyzed. To avoid bias, bacteriocyte counting and size determination were performed by three researchers in a blinded fashion.

## Results

### Life History Traits of Aphids Reared on a Tyr/Phe Depleted Artificial Diet

**FIGURE 2:**
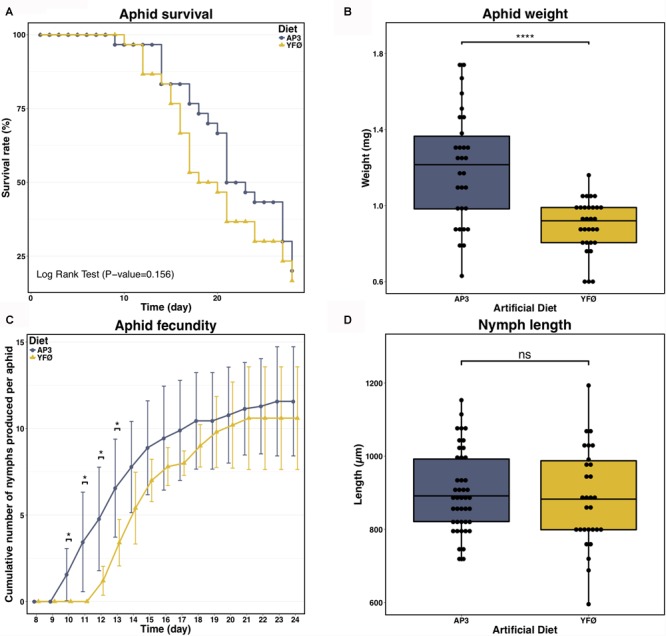
Impact of the tyrosine and phenylalanine depleted diet on aphid performances. **(A)** Survival analysis of *A. pisum* reared on two artificial diets: a complete diet (AP3, blue) and a Tyr/Phe depleted diet (YFØ, yellow). Each diet group was composed of 30 aphids. Data were analyzed using Log Rank Test. **(B)** Effects of the YFØ diet on aphid weight 7 days after the transfer on artificial diets. Each diet group was composed of 30 aphids. Data were analyzed using Welch’s *t*-test and significant differences are indicated with asterisks (^∗∗∗∗^*P* ≤ 0.0001). **(C)** Cumulative number of progeny laid per aphid reared on the YFØ and AP3 diets. Results are reported as means (±SD) of 10 isolated individuals per diet. Data were analyzed by a Welch’s *t*-test and significant differences are indicated with asterisks (^∗^*P* ≤ 0.05). **(D)** Effects of the YFØ diet on the size of produced nymphs. A total of 43 and 29 nymphs were measured for the AP3 and YFØ diet, respectively. Data were analyzed using Student’s *t*-test (ns, not significant).

Different parameters of *A. pisum* performance were recorded as metrics of aphid fitness: survival, adult weight and morphology, number of nymphs produced by the treated parthenogenetic mothers, timing of nymph production and nymph length and morphology. Despite slight differences, aphid survival was not significantly different when aphids are reared on a Tyr/Phe depleted diet (YFØ) compared to the standard artificial diet AP3 (Figure 2A, *P* = 0.156, Log Rank test). No remarkable difference in aphid size or morphology was detected. The lack of Tyr/Phe in the aphid diet did, however, induce a clear difference in aphid weight 7 days after the transfer on artificial diet (Figure 2B, *P* < 0.0001, two-sample Welch’s *t*-test) indicative of an impact on aphid physiology. This difference was also reflected in the fecundity of the parthenogenetic mothers (Figure 2C). Even if there was no significant difference in number of produced nymphs observed at the end of the laying period between aphids reared on the two diets (*P*_D24_ = 0.6212, Mann–Whitney *U* test), we observed that YFØ aphids started their laying period (Day 12) 2 days later than AP3 aphids (Day 10). This resulted in a lower number of nymphs produced by the YFØ aphids from Day 10–13 (*P*_D10_ = 0.032, *P*_D11_ = 0.015, *P*_D12_ = 0.021, *P*_D13_ = 0.014, Mann–Whitney *U* tests). However, YFØ aphids rapidly reduced the gap with AP3 aphids in the following days. Adult weight differences are probably due to the fact that 7-day-old AP3 aphids hold older (and thus more advanced and bigger) parthenogenetic embryos than YFØ aphids since the former are closer to starting their laying period. Hence, YFØ aphids physiologically require additional time to start parthenogenetic embryo production, but once the laying period has started there is no difference with AP3 aphids in reproduction. Furthermore, all progeny is normal, devoid of morphological defects and with body lengths of nymphs of both conditions not significantly different (Figure 2D, *P* = 0.544, two-sample Student’s *t*-test).

In summary, despite variations in aphid weight and onset of laying period, the overall fitness of *A. pisum* reared on the YFØ diet does not differ substantially from aphids reared on a complete artificial diet. In addition, the observation that the physiological response to Tyr/Phe nutritional stress happens rapidly suggests the presence of robust cellular and molecular strategies to control metabolic homeostasis of the pea aphid and its endosymbiont when facing a nutritional constraint.

### Bacteriocytes but Not Gut Show a Prominent Transcriptional Response to Nutritional Stress

In a time course design, aphids were collected at seven time points following their transfer on the AP3 and YFØ artificial diets. We isolated the two tissues and analyzed transcriptomes of the gut for the seven time points and of the bacteriocytes for the last four time points, due to technical limitations preventing the analysis on the three earlier time points (Figure 1).

To check overall data quality, we first performed a PCA of normalized expression values for all the 24 011 genes of each sample in all tissues and diets. Using this approach, we were able to clearly classify the data in the two tissues analyzed (Supplementary Figure S2) thereby confirming the quality of the data. In addition, the expression of seven *A. pisum* genes belonging to different functional classes was monitored using qRT-PCR to validate the microarray data (Supplementary Table S2). qRT-PCR assays were performed on the same RNA samples used for the microarray experiment (all time points from the gut and bacteriocytes tissues of AP3 and YFØ aphids). A Pearson’s coefficient of determination of 0.91 (*P* < 2.2 × 10^-16^) was observed between the qRT-PCR and microarray datasets showing a high degree of reliability for the microarray results (Supplementary Figure S3 and Supplementary Table S3).

**FIGURE 3:**
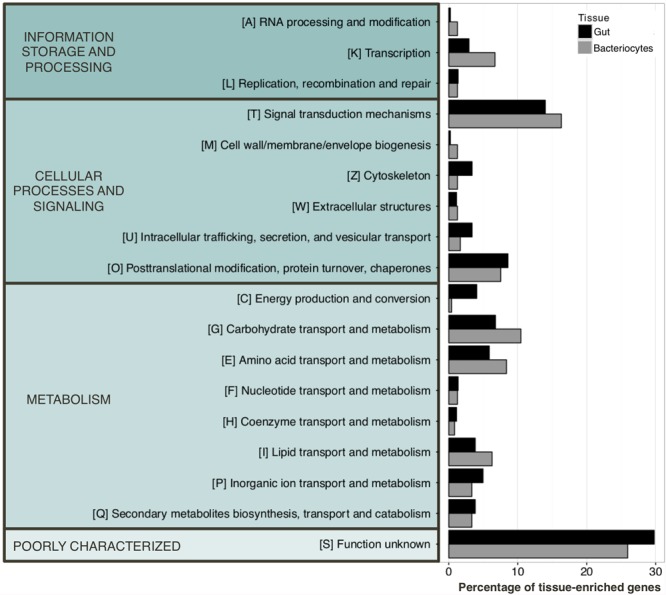
eggNOG classification of the tissue-enriched genes. Genes were defined as being tissue-enriched using a fourfold change threshold in the gut/bacteriocyte contrast. 1 079 and 638 genes were categorized as tissue specific in aphid gut and bacteriocytes, respectively.

We next identified gut- and bacteriocyte-enriched genes using data from all time points of aphid samples reared on the complete AP3 artificial diet. Genes were considered tissue-enriched using a fourfold change threshold in the gut/bacteriocyte contrast. We identified 1 079 genes mainly expressed in the gut while 638 were categorized as bacteriocyte-enriched (Supplementary Table S4). Analysis of the potential function of these genes is represented using the eggNOG classification (Huerta-Cepas et al., 2016) in Figure 3. Based on this classification, we observed that bacteriocyte-enriched genes were mainly involved in transcriptional activity, signal transduction mechanisms, and carbohydrate, amino acid and lipid transport and metabolism. The latter highlighting the pivotal role of the bacteriocytes in the symbiotic relationship between pea aphids and their endosymbiotic bacteria *Buchnera*. Transcriptional profiling of aphid gut identified genes associated with energy production and conversion, cytoskeleton, intracellular trafficking, secretion and vesicular transport are in line with its role in nutrient absorption. Note that a large proportion of genes for the two tissues are poorly annotated and were therefore categorized in a class named “function unknown.”

Differentially expressed genes from YFØ aphids compared to AP3 aphids were identified using a one-way between groups ANOVA (Limma) applied at each time point independently. Of the 10 343 transcripts analyzed, 857 (8.29%) were identified as being significantly differentially expressed during nymphal development of the pea aphid reared on a Tyr/Phe depleted diet. Strikingly, most of the transcriptional changes induced by Tyr/Phe nutritional stress occurred in bacteriocytes (771 DEGs) whereas limited transcriptomic changes were observed in gut samples (89 DEGs) (Table 1 and Supplementary Table S5). These differences in transcriptional response to nutritional stress define the bacteriocytes as a prominent mediator of metabolic homeostasis.

**Table 1:** Number of differentially expressed genes in aphids reared on a Tyr/Phe depleted diet (YFØ), in comparison to the control diet (AP3), for each tissue and each time-point.

	Day of collection	D0	D1	D2	D3	D4	D5	D7
	Time on artificial diet	12 h	1 day	2 days	3 days	4 days	5 days	7 days
Gut	Down-regulated genes	1	2	55	1	6	3	1
	Up-regulated genes	3	0	8	0	1	4	8
Bacteriocytes	Down-regulated genes				312	3	48	50
	Up-regulated genes				262	3	114	14

To characterize the role of the two tissues in the response to the nutritional stress and provide insight into the functional roles of each list of DEGs, an enrichment analysis of the functional gene classes based on the GO annotation was performed. We first performed the GO analysis using the pea aphid annotation. However, given the low number of DEGs associated with a GO term, only few and mostly high-level GO terms were identified (Supplementary Table S6). This is a problem frequently encountered with ontology-based enrichment studies in arthropods due to the large number of proteins without homologs and/or of unknown function [discussed in Wybouw et al. (2015)]. Thus, given the more extensive functional annotation of genes in *D. melanogaster*, the GO analysis was carried out using the putative *D. melanogaster* homologs for each differentially expressed *A. pisum* gene (Supplementary Tables S7, S8). While the GO analysis on the pea aphid annotation retrieved only 372 unique GO terms from the 857 different DEGs, the *D. melanogaster* GO analysis encompassed 1 978 unique GO terms. This analysis of the functional gene classes revealed that no significant GO term related to biological process, molecular function or cellular component was enriched for any of the time points sampled from the gut tissue (Supplementary Table S8) thus confirming the limited role of this tissue under Tyr/Phe nutritional stress. Conversely, this analysis showed the importance of the bacteriocytes with a total of 98 significantly enriched GO terms (Supplementary Table S8).

### Tyr/Phe Biosynthesis Is Not Regulated in the Bacteriocytes

Given that bacteriocytes together with *B. aphidicola* are essential for the production of tyrosine and phenylalanine, we first hypothesized that the expression of the genes involved in their biosynthesis would be altered to accommodate for the lack of the amino acids. To test this hypothesis, we analyzed all identified bacteriocyte DEGs using the functional annotation available in the ArthropodaCyc database (Baa-Puyoulet et al., 2016), which also contains the global reconstruction of the metabolic network of the pea aphid.

The most striking result was the absence of genes related to Tyr and Phe biosynthesis among the bacteriocyte DEGs (Table 2), despite the tyrosine biosynthetic pathway being crucial for the symbiotic metabolism of the pea aphid and *B. aphidicola* (Wilson et al., 2010) and for normal parthenogenetic development of *A. pisum* (Rabatel et al., 2013; Simonet et al., 2016b). Additionally, none of the amino acid transporters expected to have an important functional role in bacteriocytes (Price et al., 2014) was differentially regulated (Table 2). The fact that none of the genes encoding for the Tyr/Phe biosynthetic enzymes and amino acid transporters was differentially expressed in the bacteriocytes, suggests that *A. pisum* uses an alternative strategy to adapt its physiology to this nutritional constraint.

**Table 2:** Differential expression in bacteriocytes of selected genes involved in Tyr/Phe biosynthesis and amino acids transport between aphids reared on a depleted Tyr/Phe artificial diet (YFØ) and aphids reared on a complete artificial diet (AP3).

Transcript name	Description	D3		D4		D5		D7	
		log_2_FC	adj. *P*-value	log_2_FC	adj. *P*-value	log_2_FC	adj. *P*-value	log_2_FC	adj. *P*-value
**Tyr/Phe biosynthesis**									
*ACYPI000044-RA*	Aspartate transaminase	–0.1033	0.7988	–0.1562	0.9998	0.2319	0.6522	–0.0141	0.9940
*ACYPI003009-RA*	Aspartate transaminase	–0.0697	0.9197	–0.1016	0.9998	0.2219	0.8255	0.0262	0.9930
*ACYPI004243-RA*	Aspartate transaminase	0.3700	0.8505	0.7712	0.9998	–1.8012	0.2973	–1.6571	0.5150
*ACYPI006213-RA*	Aspartate transaminase	–0.1955	0.6242	–0.0210	0.9998	0.2946	0.5891	–0.3008	0.7465
*ACYPI007803-RA*	Phenylalanine hydroxylase	–0.3444	0.4518	0.3296	0.9998	0.2917	0.7089	0.0855	0.9830
**Amino acid transport**									
*ACYPI000536-RA*	Bacteriocyte amino acid transporter	–0.3052	0.5917	0.0228	0.9998	0.2186	0.8535	–0.0973	0.9830
*ACYPI000550-RA*	Bacteriocyte amino acid transporter	–0.2062	0.6387	–0.0122	0.9998	–0.0181	0.9927	–0.1004	0.9779
*ACYPI001018-RA*	Bacteriocyte amino acid transporter	–0.1246	0.6685	–0.0154	0.9998	0.1390	0.7791	–0.0383	0.9865
*ACYPI008904-RA*	Bacteriocyte amino acid transporter	–0.9696	0.4189	–0.2541	0.9998	1.3752	0.3617	–0.2160	0.9839
*ACYPI008971-RA*	Bacteriocyte amino acid transporter	0.3717	0.4871	–0.2028	0.9998	0.7704	0.1819	0.0784	0.9861

*The full list of differentially expressed genes can be retrieved from Supplementary Table S5.*

### Transcriptional Reprogramming of the Bacteriocytes

In bacteriocytes, the main transcriptional changes (574 DEGs, 74.4%) were observed 3 days after the transfer on the artificial diet whereas only 6 (0.78%), 164 (21.3%), and 65 (8.43%) were detected at Day 4, 5, and 7, respectively (Table 1). Among these DEGs, only 34 were shared between two or three time points, whereas no DEG was shared between all four time points (Supplementary Table S5).

Interestingly, the GO enrichment analysis of the bacteriocyte DEGs revealed a temporal sequence of transcriptional events allowing the *A. pisum*/*B. aphidicola* symbiotic system to cope with Tyr/Phe deprivation in the food.

**FIGURE 4:**
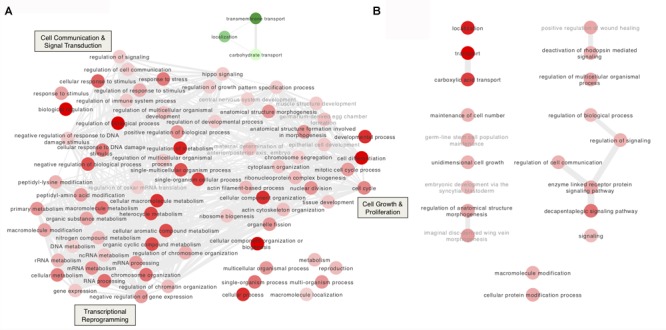
Gene Ontology (GO) enrichment analysis of the differentially expressed genes in bacteriocytes from aphids reared on the tyrosine and phenylalanine depleted diet. **(A,B)** Interactive graphs of the GO biological processes that are differentially expressed at Day 3 **(A)** and at Day 5 **(B)**. The thickness of the edges corresponds to the number of shared genes. Red and green indicate up- and down-regulation, respectively. The color density of the nodes is proportional to the FDR adjusted *P*-values. Irrelevant GO terms for the bacteriocyte cell were labeled in gray.

The large proportion (28.6%) of up-regulated genes related to “chromatin remodeling,” “histone modifications,” and “transcription factors” at Day 3 is consistent with the large number of DEGs at this time point (Figure 4A and Supplementary Tables S7, S8). Chromatin remodelers are known to regulate nucleosome dynamics to gate access to the underlying DNA for replication, repair and transcription (Petty and Pillus, 2013). Among the up-regulated genes involved in chromatin remodeling, putative *A. pisum* homologs (*ACYPI004047* and *ACYPI008655*) of the *ISWI D. melanogaster* gene were identified as well as of the *maleless* (*ACYPI002202*) and *msl-3* (*ACYPI000966*) genes (Table 3). The *ISWI* gene encodes a subunit of the nucleosome remodeling factor (Tsukiyama et al., 1995). The *maleless* and *msl-3* genes were first characterized as core members of the chromatin remodeling male specific lethal (MSL) complex (Kuroda et al., 1991; Sural et al., 2008), but *maleless* is also known to play a role in other chromatin remodeling pathways (Cugusi et al., 2015). Consistent with chromatin remodeling, several “histone modification”-related genes were up-regulated at Day 3 (Table 3) among which three putative homologs (*ACYPI003204, ACYPI006180*, and *ACYPI007884*) of the histone deacetylase 1 (*HDAC1*). Histone acetylation and deacetylation are dynamic processes with a key role in gene expression regulation by relaxing or compacting chromatin structure (Haberland et al., 2009). Together with chromatin remodeling and histone modification-associated genes, 23 transcription factors were differentially expressed at Day 3 (Table 3) suggesting that distinct transcriptional programs are activated following Tyr/Phe stress.

**Table 3:** Differential expression in bacteriocytes at Day 3 of selected genes likely involved in chromatin remodeling, histone modifications, and regulation of gene expression.

Transcript name	*Drosophila melanogaster* gene name	*D. melanogaste*r gene ID	Description	log_2_FC	adj. *P*-value
**Chromatin remodeling**					
*ACYPI004047-RA*	*ISWI*	FBgn0011604	Chromatin-remodeling complex ATPase chain ISWI	0.9220	0.0140
*ACYPI008655-RA*	*ISWI*	FBgn0011604	Chromatin-remodeling complex ATPase chain ISWI	1.2261	0.0457
*ACYPI002202-RA*	*mle*	FBgn0002774	ATP-dependent RNA helicase A	1.8789	0.0313
*ACYPI000966-RA*	*msl-3*	FBgn0002775	Male-specific lethal	0.6669	0.0254
**Histone modifications**					
*ACYPI003204-RA*	*HDAC1*	FBgn0015805	Histone deacetylase	0.7026	0.0460
*ACYPI006180-RA*	*HDAC1*	FBgn0015805	Histone deacetylase	0.9776	0.0105
*ACYPI007884-RA*	*HDAC1*	FBgn0015805	Histone deacetylase	0.9957	0.0075
**Transcription factors**					
*ACYPI20040-RA*	*CG4050*	FBgn0020312	Smile protein	–1.0657	0.0300
*ACYPI006800-RA*	*CG4328*	FBgn0036274	Homeobox domain; Zinc finger	–1.1680	0.0380
*ACYPI008133-RA*	*fd64A*	FBgn0004895	Forkhead domain 64A	–1.2241	0.0339
*ACYPI38042-RA*	*grau*	FBgn0001133	Grauzone	–0.6974	0.0488
*ACYPI25052-RA*	*ham*	FBgn0045852	Hamlet	–1.4526	0.0255
*ACYPI48767-RA*	*hng1*	FBgn0034599	Hinge1	–0.9515	0.0320
*ACYPI41460-RA*	*nab*	FBgn0259986	NAB domain	–1.7592	0.0138
*ACYPI005162-RA*	*pnr*	FBgn0003117	Pannier	–1.2368	0.0125
*ACYPI000963-RA*	*rx*	FBgn0020617	Retinal homeobox	–1.4330	0.0408
*ACYPI005588-RA*	*TH1*	FBgn0039298	Negative elongation factor C/D	–0.7142	0.0295
*ACYPI003817-RA*	*aatf*	FBgn0031851	Apoptosis antagonizing TF	0.9469	0.0125
*ACYPI008254-RA*	*alh*	FBgn0261238	Alhambra	0.7815	0.0112
*ACYPI001952-RA*	*atf6*	FBgn0033010	Basic-leucine zipper TF-like	1.5478	0.0245
*ACYPI39596-RA*	*atf6*	FBgn0033010	Basic-leucine zipper TF-like	1.6757	0.0041
*ACYPI47906-RA*	*atf6*	FBgn0033010	Basic-leucine zipper TF-like	0.6290	0.0338
*ACYPI46438-RA*	*CG32813*	FBgn0052813	Myb/SANT-like DNA-binding domain	1.0965	0.0240
*ACYPI003796-RA*	*da*	FBgn0267821	Daughterless	1.3464	0.0458
*ACYPI51211-RA*	*GATAd*	FBgn0032223	GATA transcription factor	0.7572	0.0082
*ACYPI30875-RA*	*onecut*	FBgn0028996	Onecut	0.8380	0.0245
*ACYPI008477-RA*	*pzg*	FBgn0259785	putzig-like	1.2298	0.0116
*ACYPI009169-RA*	*pzg*	FBgn0259785	putzig-like	1.4285	0.0055
*ACYPI39352-RA*	*pzg*	FBgn0259785	putzig-like	1.3891	0.0272
*ACYPI008724-RA*	*rept*	FBgn0040075	Reptin	0.8225	0.0281

*The full list of differentially expressed genes can be retrieved from Supplementary Tables S7, S8.*

### Aphids Cope With Tyr/Phe Deprivation by Increasing Bacteriocyte Number and Changing Their Phenotype

Among the differentially expressed transcription factors, three putative homologs (*ACYPI008477, ACYPI009169*, and *ACYPI39352*) of the *putzig D. melanogaster* gene were identified (Table 3). The *putzig* gene encodes a Zn-finger protein (Kugler and Nagel, 2007) belonging to a large multi-protein complex that includes the TATA-box-binding-protein-related factor 2 (TRF2) and the DNA-replication related element binding factor (DREF) (Hochheimer et al., 2002). The TRF2/DREF complex has been associated with the transcriptional regulation of replication-related genes and consists of more than a dozen proteins including several known chromatin-remodeling components such as the ISWI protein (Hochheimer et al., 2002). Accordingly, *putzig* acts as a key regulator of cell proliferation through the positive regulation of cell cycle and replication-related genes (Kugler and Nagel, 2007). The presence of up-regulated genes associated with cell proliferation in YFØ aphids after 3 days on artificial diet is consistent with several other overrepresented GO terms at this time point which were related to the cell cycle including DNA replication, cell growth and cytoskeleton reorganization (Figure 4A and Supplementary Table S8). Consistent with the cell proliferation activation, we identified, at Day 3, several up-regulated helicases involved in DNA replication and associated DNA repair processes (Table 4) such as the *mcm3* (*ACYPI005644*) as well as *mcm4* (*ACYPI004569*) genes belonging to the minichromosome maintenance (MCM) complex (Su et al., 1996). Furthermore, the *mus301* (*ACYPI004507*), *mus304* (*ACYPI008670*), and *blm*/*mus309* (*ACYPI005125*) genes involved in the DNA damage checkpoint pathways (Brodsky et al., 2000; Adams et al., 2003; McCaffrey et al., 2006) were overexpressed (Table 4).

**Table 4:** Differential expression in bacteriocytes at Day 3 of selected genes likely involved in DNA replication, cell growth and proliferation.

Transcript name	*Drosophila melanogaster* gene name	*D. melanogaste*r gene ID	Description	log_2_FC	adj. *P*-value
**DNA replication**					
*ACYPI005644-RA*	*mcm3*	FBgn0024332	DNA replication licensing factor MCM3	1.2754	0.0279
*ACYPI004569-RA*	*mcm4*	FBgn0015929	DNA replication licensing factor MCM4	1.0700	0.0312
*ACYPI004507-RA*	*mus301*	FBgn0002899	Mutagen-sensitive 301	0.8565	0.0417
*ACYPI008670-RA*	*mus304*	FBgn0002901	Mutagen-sensitive 304	1.3449	0.0480
*ACYPI005125-RA*	*blm*	FBgn0002906	Bloom syndrome helicase	0.8424	0.0342
**Hippo pathway**					
*ACYPI006463-RA*	*ft*	FBgn0001075	Fat	0.8877	0.0437
*ACYPI005965-RA*	*jub*	FBgn0030530	Ajuba	1.2951	0.0259
*ACYPI004206-RA*	*brm*	FBgn0000212	Brahma	0.5811	0.0170
**Cytoskeleton organization**					
*ACYPI007969-RA*	*asp*	FBgn0000140	Abnormal spindle	1.6541	0.0259
*ACYPI008993-RA*	*CG5023*	FBgn0038774	Putative actin binding, contains calponin domain	3.4986	0.0034
*ACYPI001560-RA*	*jv*	FBgn0263973	Javelin	1.6695	0.0448
*ACYPI003378-RA*	*kel*	FBgn0001301	Kelch	2.2605	0.0460
*ACYPI20904-RA*	*kel*	FBgn0001301	Kelch	2.5349	0.0268
*ACYPI21390-RA*	*kel*	FBgn0001301	Kelch	2.2655	0.0041
*ACYPI37466-RA*	*kel*	FBgn0001301	Kelch	1.7635	0.0268
*ACYPI006305-RA*	*msp300*	FBgn0261836	Muscle-specific protein 300 kDa	0.9898	0.0316
*ACYPI001001-RA*	*prm*	FBgn0003149	Paramyosin	3.6765	0.0368
*ACYPI003771-RA*	*siz*	FBgn0026179	Schizo	1.0640	0.0378
*ACYPI002580-RA*	*slik*	FBgn0035001	Sterile20-like kinase	0.7362	0.0422
*ACYPI008180-RA*	*sls*	FBgn0086906	Titin	1.8998	0.0483
*ACYPI008521-RA*	*tn*	FBgn0265356	Thin	2.9891	0.0170
*ACYPI007134-RA*	*tw*	FBgn0086368	Twisted	0.9822	0.0322

*The full list of differentially expressed genes can be retrieved from Supplementary Tables S7, S8.*

In addition to cell cycle and proliferation-associated genes, we also obtained further evidence indicative of cell proliferation and growth. Three genes belonging to the Hippo pathway were up-regulated in YFØ aphids at Day 3 (Table 4). Specifically, we have identified the putative *A. pisum* homologs of the *D. melanogaster* genes: *fat* (*ACYPI006463*), a receptor of the Hippo signaling pathway (Feng and Irvine, 2007), *jub* (*ACYPI005965*), which inhibits activation of this pathway (Thakur et al., 2010), and *brahma* (*ACYPI004206*), a major subunit of a chromatin-remodeling complex promoting the transcription of cell proliferation related genes (Zhu et al., 2015). Furthermore, 14 genes associated with actin cytoskeleton organization were differentially expressed in YFØ aphids at Day 3 (Figure 4A, Table 4, and Supplementary Table S8), suggestive of cytoskeletal reorganization as is observed with cell proliferation and growth. Specifically, we have identified the putative *A. pisum* homolog of *slik* (*ACYPI002580*) which has been demonstrated to stimulate cell proliferation in *Drosophila* (Hipfner and Cohen, 2003).

**FIGURE 5:**
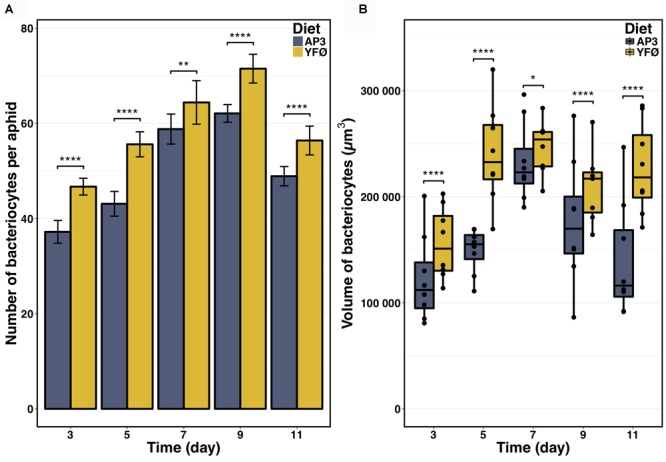
Impact of the tyrosine and phenylalanine depleted diet on aphid bacteriocytes. **(A)** Number of bacteriocytes per aphid. Results are reported as means (±SD) of 10 isolated individuals per diet. Data were analyzed using Welch’s *t*-test and significant differences are indicated with asterisks (^∗∗^*P* ≤ 0.01, ^∗∗∗∗^*P* ≤ 0.0001). **(B)** Volume of bacteriocytes. Results are displayed as box plots where central lines represent the medians, boxes comprise the 25–75 percentiles and whiskers denote the range; *n* > 7 aphids per time point, for a total number of more than 2 600 bacteriocytes isolated and analyzed. Data were analyzed using Welch’s *t*-test and significant differences are indicated with asterisks (^∗^*P* ≤ 0.05, ^∗∗∗∗^*P* ≤ 0.0001).

At Day 5 after transfer on artificial diet, the GO terms “decapentaplegic signaling pathway,” “unidimensional cell growth,” and “maintenance of cell number” were also significantly enriched (Figure 4B and Supplementary Table S8) thus providing further support for cell proliferation and growth being part of the *A. pisum* response to accommodate for a Tyr/Phe depleted food source. Therefore, to provide further experimental support for this notion, we determined number and volume of bacteriocytes from AP3 and YFØ aphids. In line with the GO analysis, we observed a significantly increased number of bacteriocytes in YFØ aphids compared to AP3 aphids from Day 3 to Day 11 (Figure 5A) coupled with a greater volume (Figure 5B).

### The Transcriptional Response of Bacteriocytes to Tyr/Phe Deprivation Is Indicative of Stress Response, Metabolic Slowdown and Adjusted Transport of Biomolecules

In addition to changes in cell proliferation and size, the transcriptional reprogramming of the bacteriocytes also involves additional processes (Figure 4 and Supplementary Table S8). The “response to stress” GO term encompasses 36 up-regulated genes at Day 3 belonging to the previously identified signaling pathways as well as the Janus Kinase (JAK)/Signal Transducer and Activator of Transcription (STAT) pathway with the presence of the *domeless* gene (*ACYPI40957*), the transmembrane receptor for signaling ligands in the cytokine family (Table 5). The conserved JAK/STAT signaling pathway transmits information received from extracellular polypeptide signals such as growth factors and cytokines, through specific transmembrane receptors, directly to target gene promoters in the nucleus, providing a mechanism for transcriptional regulation without second messengers (Aaronson and Horvath, 2002). JAK/STAT is known to regulate growth and the competitive status of proliferating cells (Mukherjee et al., 2005; Rodrigues et al., 2012) which is consistent with the previous observations.

**Table 5:** Differential expression in bacteriocytes of selected genes likely involved in stress response and sugar metabolism.

Day of collection	Transcript name	*Drosophila melanogaster* gene name	*D. melanogaste*r gene ID	Description	log_2_FC	adj. *P*-value
**JAK/STAT pathway**						
Day 3	*ACYPI40957-RA*	*dome*	FBgn0043903	Cytokine receptor	2.3755	0.0320
**Carbohydrate transport**						
Day 3	*ACYPI004517-RA*	*CG1213*	FBgn0037387	Sugar/inositol transporter	–0.8784	0.0292
Day 3	*ACYPI002572-RA*	*CG3168*	FBgn0029896	Major facilitator, sugar transporter-like	–1.1585	0.0342
Day 3	*ACYPI008900-RA*	*CG4797*	FBgn0034909	Sugar transporter	–1.8005	0.0119
Day 3	*ACYPI003707-RA*	*CG7272*	FBgn0036501	SWEET sugar transporter	–0.9714	0.0320
Day 3	*ACYPI004354-RA*	*CG8249*	FBgn0034141	Sugar/inositol transporter	–1.1501	0.0029
Day 3	*ACYPI008852-RA*	*csat*	FBgn0024994	Nucleotide-sugar transporter	–0.9734	0.0121
Day 3	*ACYPI005975-RA*	*glut1*	FBgn0264574	Glucose transporter	–0.9748	0.0266
Day 3	*ACYPI29278-RA*	*tret1-1*	FBgn0050035	Trehalose transporter 1-1	–1.6679	0.0230
**Glycolysis**						
Day 5	*ACYPI001449-RA*	*rgk1*	FBgn0264753	GTP-binding protein RAD	1.8765	0.0119
Day 7	*ACYPI001449-RA*	*rgk1*	FBgn0264753	GTP-binding protein RAD	1.9586	0.0214

*The full list of differentially expressed genes can be retrieved from Supplementary Tables S7, S8.*

Additionally, 3 days after transfer on artificial diet, eight carbohydrate transporters were down-regulated in YFØ aphids (Figure 4A, Table 5, and Supplementary Tables S7, S8). We also observed a continued overexpression of the putative *A. pisum* homolog (*ACYPI001449*) of RRAD, a small GTPase belonging to the RGK family. It has been demonstrated that this GTPase inhibits aerobic glycolysis in human cells (Wang et al., 2014; Shang et al., 2016). In addition to the carbohydrate transporters, the most enriched GO term among the down-regulated genes was also related to transport function (Figure 4A and Supplementary Table S8) suggesting a fine regulation of bacteriocyte transporter expression in YFØ aphids.

## Discussion

Nutritional symbiosis is an important factor allowing insects to feed on specialized diets and underpinning their evolutionary and ecological success. In phloem-feeding hemipterans, the close interaction with their symbiotic partners allows these insects to thrive in ecological niches associated with an unbalanced diet of plant phloem sap. Besides its nutritionally unbalanced composition, the amino acid profile of phloem sap also shows considerable spatial and temporal variability thus presenting important challenges to sap-sucking insects. This study aimed to improve our understanding of how the hemipteran *A. pisum* and its mutualist endosymbiont accommodate to this ever-changing dietary resource.

**FIGURE 6:**
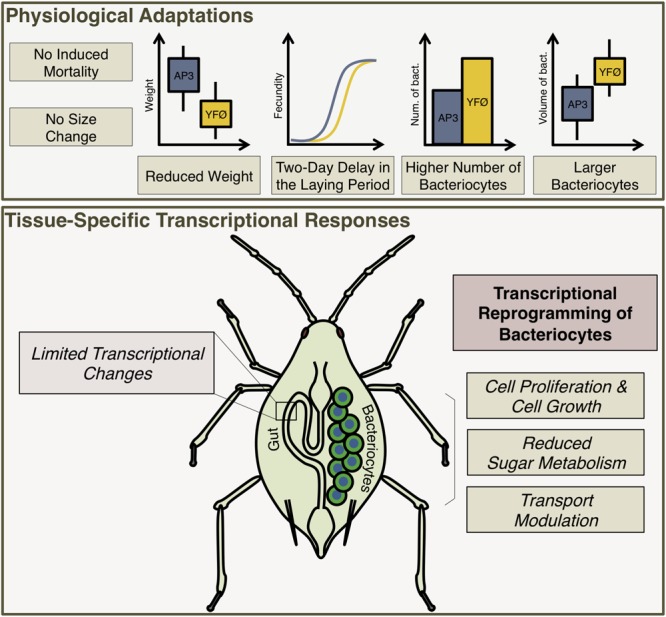
A proposed model for the response of the pea aphid to a tyrosine and phenylalanine depleted diet.

First, our results revealed remarkable physiological plasticity of the *A. pisum*/*B. aphidicola* symbiotic system to cope with changes in food source, i.e., with Tyr and Phe deprivation. This nutritional stress did not affect aphid survival and we only observed limited weight reduction and a 2-day delay in onset of laying without effect on fecundity (Figures 2, 6). This is not a general effect of lack of dietary amino acids, as depletion of other amino acids (e.g., leucine) from the AP3 diet can result in stunted aphid growth (80% reduction in size) (Viñuelas et al., 2011). Based on this, we conclude that dietary Tyr and Phe are not important for aphid survival, that they are required for normal growth but less so than Leu, and that they are essential to attain normal reproductive performance. Second, we identified the mechanisms that could be responsible for the tolerance of aphids to the depletion of these two amino acids (summarized in Figure 6).

By means of a detailed time-course analysis of *A. pisum* gene expression on a genome-wide scale, we found that bacteriocytes show very prominent changes in gene expression upon Tyr/Phe depletion, contrary to the gut where only minor changes are seen. Interestingly, neither bacteriocytes nor gut showed transcriptional changes in Tyr/Phe biosynthetic pathway or amino acid transporter genes to compensate for the deprivation of these two amino acids. Even though we cannot exclude changes of expression in these pathways in other aphid tissues, the major changes were expected in the gut, which acts as a metabolic hub in insects, and in the bacteriocytes, which have a central role in housing and maintaining endosymbiotic bacteria (Douglas, 2014) and in symbiotic amino acid metabolism and transport (Nakabachi et al., 2005; Price et al., 2014). We also cannot completely exclude that the observed lack of induction of Tyr/Phe biosynthetic pathway or amino acid transporter genes is due to the early sampling of these two tissues (collected 1–7 days after the beginning of the nutritional stress) and higher induction being possible at later stages of aphid development. However, we believe this is less likely because the highest needs for phenylalanine and tyrosine have been demonstrated to be situated during aphid nymphal development, which is almost complete at Day 7 in our experimental conditions (Rabatel et al., 2013; Simonet et al., 2016b). Finally, it is unlikely that the differences observed in bacteriocyte transcriptomes are caused indirectly by the observed reduced growth and delay in fecundity for the following reasons: (i) we observed developmental stage transitions at the same moments in our time course analysis for both AP3 and YFØ aphids; (ii) differences in developmental timing would also be expected to show in the gut, which we did not observe. Therefore, we argue that the differences seen in bacteriocyte gene expression are due to the nutritional stress imposed by the absence of Tyr/Phe in the artificial diet. The observed bacteriocyte response to metabolic changes with massive transcriptional reprogramming points to a more complex physiological role of these specialized cells central to symbiosis.

Our results indicate that bacteriocytes use alternative strategies to cope with metabolic stress different from the classic autophagy-based ones (Mizushima and Klionsky, 2007). Instead of triggering cell death (our dataset does not show induction of apoptotic and autophagy-related pathways under nutritional stress), we discovered that aphids display a defined temporal sequence of molecular events that includes an extensive transcriptional reprogramming of the bacteriocyte cells through chromatin remodeling. This involves an increased transcription of genes related to cell growth, proliferation and associated signaling pathways. Consistent with this, we observed increases in bacteriocyte cell size and in bacteriocyte cell number. We had previously shown that bacteriocyte cell numbers show dynamic changes that are coordinated with symbiont numbers throughout the aphid life cycle (Simonet et al., 2016a). Furthermore, Douglas and Dixon (1987) have shown that the number of bacteriocytes is less fixed than was initially suggested, showing that it can vary among different aphid species, and also among different morphs of the same aphid species. Finally, Hongoh and Ishikawa (1994) described an increase in bacteriocyte number in starved adult aphids provided with food. In our study, these changes in number and size of bacteriocytes would allow the aphid host to accommodate and support more endosymbionts and it could therefore represent a strategy to indirectly produce more tyrosine and phenylalanine using the precursors furnished by *B. aphidicola* to the *A. pisum*/*B. aphidicola* symbiotic system. Such strategy is consistent with previous studies that have demonstrated variations in *B. aphidicola* densities in different aphid species depending on the host plant or the availability of dietary nitrogen (Wilkinson et al., 2001, 2007; Zhang et al., 2016). We propose that the observed bacteriocyte-dependent responses are part of a dynamic response to changes in nutrition and contribute to the ecological success of these insect pests.

In parallel, we found a rapid down-regulation of genes encoding sugar transporters and genes required for sugar metabolism. Consistently, we found continued overexpression of the putative *A. pisum* homolog of RRAD, a small GTPase implicated in repressing aerobic glycolysis. The up-regulation of this gene in YFØ aphids suggests that overall sugar metabolism is repressed. Since phloem sap is a food source rich in carbohydrates, this metabolic slow-down could represent a strategy for aphids facing reduced amino acid availability in their food source to reallocate energy, through the transcriptional reprogramming previously observed, in other metabolisms and/or pathways to cope with Tyr/Phe deprivation. Furthermore, we also observed changes in the expression of other transporter genes: such transport modulation could be an additional way to change the balance of precursors in order to optimize Tyr and Phe biosynthesis and distribution in the context of a nutritional stress. It is worth noting that, as often in non-model insect genomes, numerous genes among the significantly differentially expressed lack full functional annotation thus limiting the interpretation of the results. Nonetheless, their expression pattern (e.g., enriched in bacteriocytes) suggests that (some of) these genes may play a thus far unrecognized, novel role in the response to nutritional stress, a possibility that will be explored in future work.

Altogether these results shed light on unknown aspects of the transcriptional and cellular plasticity of insect bacteriocytes. They suggest that this plasticity is essential to a phloem-feeding insect like the pea aphid to cope with the ever-changing composition of the plant phloem sap. These findings are consistent with the previously described lack of response of the symbiotic bacteria and instead show that the host, i.e., the bacteriocytes, responds in a complex manner to the deprivation of some amino acids (i.e., Tyr and Phe) in its food source. Indeed, this nutritional stress induces a rapid and extensive transcriptional reprogramming of bacteriocytes that fine-tunes physiology of these cells and leads to changes in cell size that can possibly be involved in accommodating larger numbers of endosymbionts. By extension, equivalent responses may be present in other biological systems that depend on nutritionally unbalanced diets for their growth and reproduction, a hypothesis that remains to be tested in future research. Finally, our results on the *A. pisum*/*B. aphidicola* system raise further interesting future research perspectives that include (i) the bacteriocyte responses to deprivation of other amino acids and other nutritional/environmental challenges, (ii) the impact of additional endosymbionts on this process in multi-partner symbiotic relationships widely found in nature, and (iii) the role of bacteriocytes in nutritional homeostasis mechanisms in other aphids feeding on different host plants, in other hemipteran species (e.g., whiteflies and psyllids), and finally in symbiosis in other insect orders.

## Data Availability

The datasets generated for this study can be found in the ArrayExpress database (Accession No. E-MTAB-4456).

## Author Contributions

SC, YR, HC, GF, and FC conceived and designed the study. SC, PS, KG, GD, and FC performed the experiments. SC, NP, PS, PB-P, PC, and FC performed the data analysis. SC, NP, PS, GF, PC, and FC interpreted the results. SC, NP, PC, and FC wrote the paper with contributions from KG. All authors revised and approved the manuscript.

## Conflict of Interest Statement

The authors declare that the research was conducted in the absence of any commercial or financial relationships that could be construed as a potential conflict of interest.

## Footnotes

1 http://rest.gene-quantification.info/

## References

[B1] Aaronson D. S. Horvath C. M. (2002). A road map for those who don’t know JAK-STAT. *Science* 296 1653–1655. 10.1126/science.1071545 12040185

[B2] Adams M. D. McVey M. Sekelsky J. J. (2003). *Drosophila* BLM in double-strand break repair by synthesis-dependent strand annealing. *Science* 299 265–267. 10.1126/science.1077198 12522255

[B3] Akman Gündüz E. Douglas A. E. (2009). Symbiotic bacteria enable insect to use a nutritionally inadequate diet. *Proc. R. Soc. Lond. B* 276 987–991. 10.1098/rspb.2008.1476 19129128 PMC2664372

[B4] Altschul S. F. Gish W. Miller W. Myers E. W. Lipman D. J. (1990). Basic local alignment search tool. *J. Mol. Biol.* 215 403–410. 10.1016/S0022-2836(05)80360-2 2231712

[B5] Baa-Puyoulet P. Parisot N. Febvay G. Huerta-Cepas J. Vellozo A. F. Gabaldón T. (2016). ArthropodaCyc: a CycADS powered collection of BioCyc databases to analyse and compare metabolism of arthropods. *Database* 2016:baw081. 10.1093/database/baw081 27242037 PMC5630938

[B6] Baumann P. Baumann L. Lai C. Y. Rouhbakhsh D. Moran N. A. Clark M. A. (1995). Genetics, physiology, and evolutionary relationships of the genus *Buchnera*: intracellular symbionts of aphids. *Annu. Rev. Microbiol.* 49 55–94. 10.1146/annurev.mi.49.100195.000415 8561471

[B7] Bermingham J. Wilkinson T. L. (2010). The role of intracellular symbiotic bacteria in the amino acid nutrition of embryos from the black bean aphid, *Aphis fabae*. *Entomol. Exp. Appl.* 134 272–279. 10.1111/j.1570-7458.2009.00953.x

[B8] Brault V. Uzest M. Monsion B. Jacquot E. Blanc S. (2010). Aphids as transport devices for plant viruses. *C. R. Biol.* 333 524–538. 10.1016/j.crvi.2010.04.001 20541164

[B9] Brinza L. Viñuelas J. Cottret L. Calevro F. Rahbé Y. Febvay G. (2009). Systemic analysis of the symbiotic function of *Buchnera aphidicola*, the primary endosymbiont of the pea aphid *Acyrthosiphon pisum*. *C. R. Biol.* 332 1034–1049. 10.1016/j.crvi.2009.09.007 19909925

[B10] Brodsky M. H. Sekelsky J. J. Tsang G. Hawley R. S. Rubin G. M. (2000). mus304 encodes a novel DNA damage checkpoint protein required during *Drosophila* development. *Genes Dev.* 14 666–678. 10733527 PMC316460

[B11] Buchner P. (1965). *Endosymbiosis of Animals with Plant Microorganisms.* New-York, NY: John Wiley & Sons.

[B12] Corbesier L. Havelange A. Lejeune P. Bernier G. Périlleux C. (2001). N content of phloem and xylem exudates during the transition to flowering in *Sinapis alba* and *Arabidopsis thaliana*. *Plant Cell Environ.* 24 367–375. 10.1046/j.1365-3040.2001.00683.x

[B13] Cugusi S. Kallappagoudar S. Ling H. Lucchesi J. C. (2015). The *Drosophila helicase* maleless (MLE) is implicated in functions distinct from its role in dosage compensation. *Mol. Cell. Proteom.* 14 1478–1488. 10.1074/mcp.M114.040667 25776889 PMC4458714

[B14] Dallas P. B. Gottardo N. G. Firth M. J. Beesley A. H. Hoffmann K. Terry P. A. (2005). Gene expression levels assessed by oligonucleotide microarray analysis and quantitative real-time RT-PCR – how well do they correlate? *BMC Genomics* 6:59. 10.1186/1471-2164-6-59 15854232 PMC1142514

[B15] Dinant S. Bonnemain J.-L. Girousse C. Kehr J. (2010). Phloem sap intricacy and interplay with aphid feeding. *C. R. Biol.* 333 504–515. 10.1016/j.crvi.2010.03.008 20541162

[B16] Douglas A. E. (1993). The nutritional quality of phloem sap utilized by natural aphid populations. *Ecol. Entomol.* 18 31–38. 10.1111/j.1365-2311.1993.tb01076.x

[B17] Douglas A. E. (1996). Reproductive failure and the free amino acid pools in pea aphids (*Acyrthosiphon pisum*) lacking symbiotic bacteria. *J. Insect Physiol.* 42 247–255. 10.1016/0022-1910(95)00105-0

[B18] Douglas A. E. (2003). The nutritional physiology of aphids. *Adv. Insect Physiol.* 31 73–140.

[B19] Douglas A. E. (2006). Phloem-sap feeding by animals: problems and solutions. *J. Exp. Bot.* 57 747–754. 10.1093/jxb/erj067 16449374

[B20] Douglas A. E. (2014). The molecular basis of bacterial-insect symbiosis. *J. Mol. Biol.* 426 3830–3837. 10.1016/j.jmb.2014.04.005 24735869 PMC4385585

[B21] Douglas A. E. Dixon A. F. G. (1987). The mycetocyte symbiosis of aphids: variation with age and morph in virginoparae of *Megoura viciae* and *Acyrthosiphon pisum*. *J. Insect Physiol.* 33 109–113. 10.1016/0022-1910(87)90082-5

[B22] Dray S. Dufour A.-B. (2007). The ade4 package: implementing the duality diagram for ecologists. *J. Stat. Softw.* 22 1–20.

[B23] Dudoit S. Shaffer J. P. Boldrick J. C. (2003). Multiple hypothesis testing in microarray experiments. *Stat. Sci.* 18 71–103. 10.2307/3182872

[B24] Febvay G. Delobel B. Rahbé Y. (1988). Influence of the amino-acid balance on the improvement of an artificial diet for a biotype of *Acyrthosiphon pisum* (Homoptera, Aphididae). *Can. J. Zool.* 66 2449–2453. 10.1139/z88-362

[B25] Febvay G. Rahbé Y. Rynkiewicz M. Guillaud J. Bonnot G. (1999). Fate of dietary sucrose and neosynthesis of amino acids in the pea aphid, *Acyrthosiphon pisum*, reared on different diets. *J. Exp. Biol.* 202(Pt 19) 2639–2652. 10482723 10.1242/jeb.202.19.2639

[B26] Feng Y. Irvine K. D. (2007). Fat and expanded act in parallel to regulate growth through warts. *Proc. Natl. Acad. Sci. U.S.A.* 104 20362–20367. 10.1073/pnas.0706722105 18077345 PMC2154436

[B27] Gholami M. (2004). Grapevine phloem sap analysis: 1- sucrose, amino acids, potassium concentrations, seasonal and diurnal patterns. *Acta Hortic.* 640 143–153.

[B28] Gil R. Sabater-Munoz B. Latorre A. Silva F. J. Moya A. (2002). Extreme genome reduction in *Buchnera* spp.: toward the minimal genome needed for symbiotic life. *Proc. Natl. Acad. Sci. U.S.A.* 99 4454–4458. 10.1073/pnas.062067299 11904373 PMC123669

[B29] Girousse C. Moulia B. Silk W. Bonnemain J.-L. (2005). Aphid infestation causes different changes in carbon and nitrogen allocation in alfalfa stems as well as different inhibitions of longitudinal and radial expansion. *Plant Physiol.* 137 1474–1484. 10.1104/pp.104.057430 15778456 PMC1088336

[B30] Gramates L. S. Marygold S. J. Santos G. D. Urbano J.-M. Antonazzo G. Matthews B. B. (2017). FlyBase at 25: looking to the future. *Nucleic Acids Res.* 45 D663–D671. 10.1093/nar/gkw1016 27799470 PMC5210523

[B31] Haberland M. Montgomery R. L. Olson E. N. (2009). The many roles of histone deacetylases in development and physiology: implications for disease and therapy. *Nat. Rev. Genet.* 10 32–42. 10.1038/nrg2485 19065135 PMC3215088

[B32] Hansen A. K. Moran N. A. (2011). Aphid genome expression reveals host-symbiont cooperation in the production of amino acids. *Proc. Natl. Acad. Sci. U.S.A.* 108 2849–2854. 10.1073/pnas.1013465108 21282658 PMC3041126

[B33] Hayashi H. Chino M. (1986). Collection of pure phloem sap from wheat and its chemical composition. *Plant Cell Physiol.* 27 1387–1393.

[B34] Hayashi H. Chino M. (1990). Chemical composition of phloem sap from the uppermost internode of the rice plant. *Plant Cell Physiol.* 31 247–251. 10.1093/oxfordjournals.pcp.a077899

[B35] Hipfner D. R. Cohen S. M. (2003). The *Drosophila* sterile-20 kinase slik controls cell proliferation and apoptosis during imaginal disc development. *PLoS Biol.* 1:E35. 10.1371/journal.pbio.0000035 14624240 PMC261876

[B36] Hochheimer A. Zhou S. Zheng S. Holmes M. C. Tjian R. (2002). TRF2 associates with DREF and directs promoter-selective gene expression in *Drosophila*. *Nature* 420 439–445. 10.1038/nature01167 12459787

[B37] Hongoh Y. Ishikawa H. (1994). Changes of mycetocyte symbiosis in response to flying behavior of alatiform aphid (*Acyrthosiphon pisum*). *Zool. Sci.* 11 731–735.

[B38] Huang J.-H. Jing X. Douglas A. E. (2015). The multi-tasking gut epithelium of insects. *Insect Biochem. Mol. Biol.* 67 15–20. 10.1016/j.ibmb.2015.05.004 25982023 PMC4644519

[B39] Huerta-Cepas J. Szklarczyk D. Forslund K. Cook H. Heller D. Walter M. C. (2016). eggNOG 4.5: a hierarchical orthology framework with improved functional annotations for eukaryotic, prokaryotic and viral sequences. *Nucleic Acids Res.* 44 D286–D293. 10.1093/nar/gkv1248 26582926 PMC4702882

[B40] International Aphid Genomics Consortium (2010). Genome sequence of the pea aphid *Acyrthosiphon pisum*. *PLoS Biol.* 8:e1000313. 10.1371/journal.pbio.1000313 20186266 PMC2826372

[B41] Irizarry R. A. Hobbs B. Collin F. Beazer-Barclay Y. D. Antonellis K. J. Scherf U. (2003). Exploration, normalization, and summaries of high density oligonucleotide array probe level data. *Biostatistics* 4 249–264. 10.1093/biostatistics/4.2.249 12925520

[B42] Karley A. J. Douglas A. E. Parker W. E. (2002). Amino acid composition and nutritional quality of potato leaf phloem sap for aphids. *J. Exp. Biol.* 205 3009–3018. 12200404 10.1242/jeb.205.19.3009

[B43] Kugler S. J. Nagel A. C. (2007). putzig is required for cell proliferation and regulates notch activity in *Drosophila*. *Mol. Biol. Cell* 18 3733–3740. 10.1091/mbc.E07-03-0263 17634285 PMC1995712

[B44] Kuroda M. I. Kernan M. J. Kreber R. Ganetzky B. Baker B. S. (1991). The maleless protein associates with the X chromosome to regulate dosage compensation in *Drosophila*. *Cell* 66 935–947. 1653648 10.1016/0092-8674(91)90439-6

[B45] Maere S. Heymans K. Kuiper M. (2005). BiNGO: a Cytoscape plugin to assess overrepresentation of gene ontology categories in biological networks. *Bioinformatics* 21 3448–3449. 10.1093/bioinformatics/bti551 15972284

[B46] McCaffrey R. St Johnston D. González-Reyes A. (2006). *Drosophila* mus301/spindle-C encodes a helicase with an essential role in double-strand DNA break repair and meiotic progression. *Genetics* 174 1273–1285. 10.1534/genetics.106.058289 16888338 PMC1667076

[B47] Mizushima N. Klionsky D. J. (2007). Protein turnover via autophagy: implications for metabolism. *Annu. Rev. Nutr.* 27 19–40. 10.1146/annurev.nutr.27.061406.093749 17311494

[B48] Moran N. Baumann P. (1994). Phylogenetics of cytoplasmically inherited microorganisms of arthropods. *Trends Ecol. Evol.* 9 15–20. 10.1016/0169-5347(94)90226-7 21236755

[B49] Moran N. A. Dunbar H. E. Wilcox J. L. (2005). Regulation of transcription in a reduced bacterial genome: nutrient-provisioning genes of the obligate symbiont *Buchnera aphidicola*. *J. Bacteriol.* 187 4229–4237. 10.1128/JB.187.12.4229-4237.2005 15937185 PMC1151715

[B50] Moran N. A. Munson M. A. Baumann P. Ishikawa H. (1993). A molecular clock in endosymbiotic bacteria is calibrated using the insect hosts. *Proc. R. Soc. Lond. B* 253 167–171. 10.1098/rspb.1993.0098

[B51] Mukherjee T. Hombría J. C.-G. Zeidler M. P. (2005). Opposing roles for *Drosophila* JAK/STAT signalling during cellular proliferation. *Oncogene* 24 2503–2511. 10.1038/sj.onc.1208487 15735706

[B52] Nakabachi A. Sakazume N. Shiraki T. Hayashizaki Y. Carninci P. Ishikawa H. (2005). Transcriptome analysis of the aphid bacteriocyte, the symbiotic host cell that harbors an endocellular mutualistic bacterium, *Buchnera*. *Proc. Natl. Acad. Sci. U.S.A.* 102 5477–5482. 10.1073/pnas.0409034102 15800043 PMC555734

[B53] Perilla-Henao L. M. Casteel C. L. (2016). Vector-borne bacterial plant pathogens: interactions with hemipteran insects and plants. *Front. Plant Sci.* 7:1163. 10.3389/fpls.2016.01163 27555855 PMC4977473

[B54] Petty E. Pillus L. (2013). Balancing chromatin remodeling and histone modifications in transcription. *Trends Genet.* 29 621–629. 10.1016/j.tig.2013.06.006 23870137 PMC4408995

[B55] Pfaffl M. W. Horgan G. W. Dempfle L. (2002). Relative expression software tool (REST) for group-wise comparison and statistical analysis of relative expression results in real-time PCR. *Nucleic Acids Res.* 30:e36. 11972351 10.1093/nar/30.9.e36 PMC113859

[B56] Pfaffl M. W. Tichopad A. Prgomet C. Neuvians T. P. (2004). Determination of stable housekeeping genes, differentially regulated target genes and sample integrity: BestKeeper–Excel-based tool using pair-wise correlations. *Biotechnol. Lett.* 26 509–515. 15127793 10.1023/b:bile.0000019559.84305.47

[B57] Poliakov A. Russell C. W. Ponnala L. Hoops H. J. Sun Q. Douglas A. E. (2011). Large-scale label-free quantitative proteomics of the pea aphid-*Buchnera* symbiosis. *Mol. Cell. Proteomics* 10:M110.007039. 10.1074/mcp.M110.007039 21421797 PMC3108839

[B58] Price D. R. G. Feng H. Baker J. D. Bavan S. Luetje C. W. Wilson A. C. C. (2014). Aphid amino acid transporter regulates glutamine supply to intracellular bacterial symbionts. *Proc. Natl. Acad. Sci. U.S.A.* 111 320–325. 10.1073/pnas.1306068111 24367072 PMC3890774

[B59] Prüfer K. Stenzel U. Dannemann M. Green R. E. Lachmann M. Kelso J. (2008). PatMaN: rapid alignment of short sequences to large databases. *Bioinformatics* 24 1530–1531. 10.1093/bioinformatics/btn223 18467344 PMC2718670

[B60] Rabatel A. Febvay G. Gaget K. Duport G. Baa-Puyoulet P. Sapountzis P. (2013). Tyrosine pathway regulation is host-mediated in the pea aphid symbiosis during late embryonic and early larval development. *BMC Genomics* 14:235. 10.1186/1471-2164-14-235 23575215 PMC3660198

[B61] Rahbé Y. Digilio M. C. Febvay G. Guillaud J. Fanti P. Pennacchio F. (2002). Metabolic and symbiotic interactions in amino acid pools of the pea aphid, *Acyrthosiphon pisum*, parasitized by the braconid *Aphidius ervi*. *J. Insect Physiol.* 48 507–516. 10.1016/S0022-1910(02)00053-7 12770078

[B62] Reymond N. Calevro F. Viñuelas J. Morin N. Rahbé Y. Febvay G. (2006). Different levels of transcriptional regulation due to trophic constraints in the reduced genome of *Buchnera aphidicola* APS. *Appl. Environ. Microbiol.* 72 7760–7766. 10.1128/AEM.01118-06 17041159 PMC1694209

[B63] Ritchie M. E. Phipson B. Wu D. Hu Y. Law C. W. Shi W. (2015). limma powers differential expression analyses for RNA-sequencing and microarray studies. *Nucleic Acids Res.* 43:e47. 10.1093/nar/gkv007 25605792 PMC4402510

[B64] Rodrigues A. B. Zoranovic T. Ayala-Camargo A. Grewal S. Reyes-Robles T. Krasny M. (2012). Activated STAT regulates growth and induces competitive interactions independently of Myc, Yorkie, Wingless and ribosome biogenesis. *Development* 139 4051–4061. 10.1242/dev.076760 22992954 PMC3472591

[B65] Rychlik W. (2007). OLIGO 7 primer analysis software. *Methods Mol. Biol.* 402 35–60. 10.1007/978-1-59745-528-2_2 17951789

[B66] Sandström J. Pettersson J. (1994). Amino acid composition of phloem sap and the relation to intraspecific variation in pea aphid (*Acyrthosiphon pisum*) performance. *J. Insect Physiol.* 40 947–955. 10.1016/0022-1910(94)90133-3

[B67] Schneider J. Buness A. Huber W. Volz J. Kioschis P. Hafner M. (2004). Systematic analysis of T7 RNA polymerase based in vitro linear RNA amplification for use in microarray experiments. *BMC Genomics* 5:29. 10.1186/1471-2164-5-29 15119961 PMC419340

[B68] Shang R. Wang J. Sun W. Dai B. Ruan B. Zhang Z. (2016). RRAD inhibits aerobic glycolysis, invasion, and migration and is associated with poor prognosis in hepatocellular carcinoma. *Tumour Biol.* 37 5097–5105. 10.1007/s13277-015-4329-7 26546438

[B69] Shannon P. Markiel A. Ozier O. Baliga N. S. Wang J. T. Ramage D. (2003). Cytoscape: a software environment for integrated models of biomolecular interaction networks. *Genome Res.* 13 2498–2504. 10.1101/gr.1239303 14597658 PMC403769

[B70] Sharkey P. J. Pate J. S. (1976). Translocation from leaves to fruits of a legume, studied by a phloem bleeding technique: diurnal changes and effects of continuous darkness. *Planta* 128 63–72. 10.1007/BF00397180 24430608

[B71] Shigenobu S. Watanabe H. Hattori M. Sakaki Y. Ishikawa H. (2000). Genome sequence of the endocellular bacterial symbiont of aphids *Buchnera* sp. APS. *Nature* 407 81–86. 10.1038/35024074 10993077

[B72] Simonet P. Duport G. Gaget K. Weiss-Gayet M. Colella S. Febvay G. (2016a). Direct flow cytometry measurements reveal a fine-tuning of symbiotic cell dynamics according to the host developmental needs in aphid symbiosis. *Sci. Rep.* 6:19967. 10.1038/srep19967 26822159 PMC4731799

[B73] Simonet P. Gaget K. Parisot N. Duport G. Rey M. Febvay G. (2016b). Disruption of phenylalanine hydroxylase reduces adult lifespan and fecundity, and impairs embryonic development in parthenogenetic pea aphids. *Sci. Rep.* 6:34321. 10.1038/srep34321 27694983 PMC5046115

[B74] Simonet P. Gaget K. Balmand S. Ribeiro Lopes M. Parisot N. Buhler K. (2018). Bacteriocyte cell death in the pea aphid/Buchnera symbiotic system. *Proc. Natl. Acad. Sci. U.S.A.* 115 E1819–E1828. 10.1073/pnas.1720237115 29432146 PMC5828623

[B75] Smith J. A. C. Milburn J. A. (1980). Phloem transport, solute flux and the kinetics of sap exudation in *Ricinus communis* L. *Planta* 148 35–41. 10.1007/BF00385439 24311263

[B76] Su T. T. Feger G. O’Farrell P. H. (1996). *Drosophila* MCM protein complexes. *Mol. Biol. Cell* 7 319–329. 8688561 10.1091/mbc.7.2.319 PMC275882

[B77] Supek F. Bošnjak M. Škunca N. Šmuc T. (2011). REVIGO summarizes and visualizes long lists of gene ontology terms. *PLoS One* 6:e21800. 10.1371/journal.pone.0021800 21789182 PMC3138752

[B78] Sural T. H. Peng S. Li B. Workman J. L. Park P. J. Kuroda M. I. (2008). The MSL3 chromodomain directs a key targeting step for dosage compensation of the *Drosophila melanogaster* X chromosome. *Nat. Struct. Mol. Biol.* 15 1318–1325. 10.1038/nsmb.1520 19029895 PMC2636508

[B79] Tagu D. Dugravot S. Outreman Y. Rispe C. Simon J.-C. Colella S. (2010). The anatomy of an aphid genome: from sequence to biology. *C. R. Biol.* 333 464–473. 10.1016/j.crvi.2010.03.006 20541158

[B80] Thakur D. M. Feng Y. Jagannathan R. Seppa M. J. Skeath J. B. Longmore G. D. (2010). Ajuba LIM proteins are negative regulators of the Hippo signaling pathway. *Curr. Biol.* 20 657–662. 10.1016/j.cub.2010.02.035 20303269 PMC2855397

[B81] Tsukiyama T. Daniel C. Tamkun J. Wu C. (1995). ISWI, a member of the SWI2/SNF2 ATPase family, encodes the 140 kDa subunit of the nucleosome remodeling factor. *Cell* 83 1021–1026. 10.1016/0092-8674(95)90217-1 8521502

[B82] Viñuelas J. Febvay G. Duport G. Colella S. Fayard J.-M. Charles H. (2011). Multimodal dynamic response of the *Buchnera aphidicola* pLeu plasmid to variations in leucine demand of its host, the pea aphid *Acyrthosiphon pisum*. *Mol. Microbiol.* 81 1271–1285. 10.1111/j.1365-2958.2011.07760.x 21797941 PMC3229713

[B83] Von Dohlen C. D. Moran N. A. (2000). Molecular data support a rapid radiation of aphids in the Cretaceous and multiple origins of host alternation. *Biol. J. Linn. Soc.* 71 689–717. 10.1006/bijl.2000.0470

[B84] Wang Y. Li G. Mao F. Li X. Liu Q. Chen L. (2014). Ras-induced epigenetic inactivation of the RRAD (Ras-related associated with diabetes) gene promotes glucose uptake in a human ovarian cancer model. *J. Biol. Chem.* 289 14225–14238. 10.1074/jbc.M113.527671 24648519 PMC4022888

[B85] Wilkinson T. L. Adams D. Minto L. B. Douglas A. E. (2001). The impact of host plant on the abundance and function of symbiotic bacteria in an aphid. *J. Exp. Biol.* 204 3027–3038. 10.1007/s004420050190 11551991

[B86] Wilkinson T. L. Ishikawa H. (1999). The assimilation and allocation of nutrients by symbiotic and aposymbiotic pea aphids, *Acyrthosiphon pisum*. *Entomol. Exp. Appl.* 91 195–201. 10.1046/j.1570-7458.1999.00484.x

[B87] Wilkinson T. L. Ishikawa H. (2000). Injection of essential amino acids substitutes for bacterial supply in aposymbiotic pea aphids (*Acyrthosiphon pisum*). *Entomol. Exp. Appl.* 94 85–91. 10.1023/A:1003909127158

[B88] Wilkinson T. L. Koga R. Fukatsu T. (2007). Role of host nutrition in symbiont regulation: impact of dietary nitrogen on proliferation of obligate and facultative bacterial endosymbionts of the pea aphid *Acyrthosiphon pisum*. *Appl. Environ. Microbiol.* 73 1362–1366. 10.1128/AEM.01211-06 17158610 PMC1828675

[B89] Wilson A. C. C. (2011). Genomic revelations of a mutualism: the pea aphid and its obligate bacterial symbiont. *Cell Mol. Life Sci.* 68 1297–1309. 10.1007/s00018-011-0645-2 21390549 PMC3064905

[B90] Wilson A. C. C. Ashton P. D. Calevro F. Charles H. Colella S. Febvay G. (2010). Genomic insight into the amino acid relations of the pea aphid, *Acyrthosiphon pisum*, with its symbiotic bacterium *Buchnera aphidicola*. *Insect Mol. Biol.* 19(Suppl. 2) 249–258. 10.1111/j.1365-2583.2009.00942.x 20482655

[B91] Wilson A. C. C. Dunbar H. E. Davis G. K. Hunter W. B. Stern D. L. Moran N. A. (2006). A dual-genome microarray for the pea aphid, *Acyrthosiphon pisum*, and its obligate bacterial symbiont, *Buchnera aphidicola*. *BMC Genomics* 7:50. 10.1186/1471-2164-7-50 16536873 PMC1440324

[B92] Wilson A. C. C. Duncan R. P. (2015). Signatures of host/symbiont genome coevolution in insect nutritional endosymbioses. *Proc. Natl. Acad. Sci. U.S.A.* 112 10255–10261. 10.1073/pnas.1423305112 26039986 PMC4547219

[B93] Winter H. Lohaus G. Heldt H. W. (1992). Phloem transport of amino acids in relation to their cytosolic levels in barley leaves. *Plant Physiol.* 99 996–1004. 10.1104/pp.99.3.996 16669030 PMC1080575

[B94] Wybouw N. Zhurov V. Martel C. Bruinsma K. A. Hendrickx F. Grbiæ V. (2015). Adaptation of a polyphagous herbivore to a novel host plant extensively shapes the transcriptome of herbivore and host. *Mol. Ecol.* 24 4647–4663. 10.1111/mec.13330 26211543

[B95] Zhang Y.-C. Cao W.-J. Zhong L.-R. Godfray H. C. J. Liu X.-D. (2016). Host plant determines the population size of an obligate symbiont (*Buchnera aphidicola*) in aphids. *Appl. Environ. Microbiol.* 82 2336–2346. 10.1128/AEM.04131-15 26850304 PMC4959500

[B96] Zhu Y. Li D. Wang Y. Pei C. Liu S. Zhang L. (2015). Brahma regulates the Hippo pathway activity through forming complex with Yki-Sd and regulating the transcription of Crumbs. *Cell Signal.* 27 606–613. 10.1016/j.cellsig.2014.12.002 25496831

