# Supplementary material for: Bacteriocyte Reprogramming to Cope With Nutritional Stress in a Phloem Sap Feeding Hemipteran, the Pea Aphid Acyrthosiphon pisum
Source: Front Physiol. 2018 Oct 25;9:1498. doi: 10.3389/fphys.2018.01498 (PMC6209921; doi:10.3389/fphys.2018.01498)
Supplement: Supplementary file 2 [file Table_2.PDF]

**Table S2.** Oligonucleotide primers used for qRT-PCR.

| ACYPI mRNA ID | Primer | Sequences | Amplicon length (bp) |
|---|---|---|---|
| *ACYPI000653* | Forward (F) | 5'- GGT-ATA-GCA-TTC-GGA-TTG-TAT -3' | 107 |
| | Reverse (R) | 5'- AGT-ATT-CCT-CCT-TCG-TGT-GG -3' | |
| *ACYPI001281* | Forward (F) | 5'- CAG-TGT-TAC-AAA-GAT-CAG-TG -3' | 190 |
| | Reverse (R) | 5'- ATA-TTC-CTC-CTT-TAT-AGC-ATA-AG -3' | |
| *ACYPI001701* | Forward (F) | 5'- ACG-GTC-TTT-GGG-CAA-TGT-TAG -3' | 130 |
| | Reverse (R) | 5'- GGC-CAC-TGG-TTT-AAT-ATC-CCT -3' | |
| *ACYPI003338* | Forward (F) | 5'- GGC-CGA-ACC-AGT-AAT-GAC -3' | 127 |
| | Reverse (R) | 5'- ATG-GTG-CCT-TTC-CGA-GAC-TGA -3' | |
| *ACYPI004647* | Forward (F) | 5'- TAT-ATT-TCT-GGA-GGT-ACA-ACA -3' | 128 |
| | Reverse (R) | 5'- TTG-GCT-ACG-TGA-CGA-T -3' | |
| *ACYPI006800* | Forward (F) | 5'- TGC-GAC-GAT-GGT-GTA-TCT-G -3' | 237 |
| | Reverse (R) | 5'- AAT-GTG-CTT-GGG-TAT-CAT-AAT -3' | |
| *ACYPI010105* | Forward (F) | 5'- CTA-TCG-GAG-GTG-ATT-GTG-T -3' | 171 |
| | Reverse (R) | 5'- TAT-TTT-CTG-TAA-TCG-GTG-GTT -3' | |
| *ACYPI010200* (RPL7) | Forward (F) | 5'- TCA-AGG-GAC-AAC-GCA-TTC -3' | 142 |
| | Reverse (R) | 5'- CAA-AGG-AAG-TTC-ATC-GCA-TAC -3' | |
