# Supplementary material for: Bacteriocyte Reprogramming to Cope With Nutritional Stress in a Phloem Sap Feeding Hemipteran, the Pea Aphid Acyrthosiphon pisum
Source: Front Physiol. 2018 Oct 25;9:1498. doi: 10.3389/fphys.2018.01498 (PMC6209921; doi:10.3389/fphys.2018.01498)
Supplement: Supplementary file 3 [file Table_3.PDF]

Table S3. Microarray data validation by qRT-PCR.

Gene	Annotation	qRT-PCR data			Microarray data		
		Time	Log_2 expression ratio YFØ/AP3	Regulation	Time	Log_2 fold change YFØ/AP3	Regulation
Bacteriocyte DEGs							
ACYPI001281	cathepsin B	D3	**-1.67**	**down**	D3	**-1.54**	**down**
		D4	**1.04**	**up**	D4	0.14	invariant
		D5	**-4.12**	**down**	D5	**-2.43**	**down***
		D7	**-2.30**	**down**	D7	**-2.84**	**down***
ACYPI003338	cysteine-type endopeptidase	D3	**-2.09**	**down**	D3	**-2.56**	**down***
		D4	-0.13	invariant	D4	-0.10	invariant
		D5	**-0.59**	**down**	D5	**-1.42**	**down**
		D7	**-1.20**	**down**	D7	**-2.44**	**down***
ACYPI004647	protein tyrosine phosphatase	D3	-0.21	invariant	D3	**-1.04**	**down**
		D4	**1.92**	**up**	D4	**1.99**	**up**
		D5	**2.27**	**up**	D5	**2.06**	**up***
		D7	**2.11**	**up**	D7	**2.69**	**up***
ACYPI006800	transcriptional regulator	D3	**-1.05**	**down**	D3	**-1.17**	**down***
		D4	-0.19	invariant	D4	0.22	invariant
		D5	**-0.57**	**down**	D5	**-1.20**	**down**
		D7	**1.63**	**up**	D7	**1.70**	**up***
Gut DEGs							
ACYPI000653	acetyl-coA transporter	D0	0.81	invariant	D0	0.04	invariant
		D1	**-1.22**	**down**	D1	**-1.60**	**down**
		D2	**-2.38**	**down**	D2	**-3.29**	**down***
		D3	1.92	**up**	D3	0.57	invariant
		D4	-0.49	invariant	D4	-0.43	invariant
		D5	**-1.35**	**down**	D5	**-2.14**	**down**
		D7	**-0.57**	**down**	D7	**-1.01**	**down**
ACYPI001281	cathepsin B	D0	0.44	invariant	D0	0.02	invariant
		D1	-0.06	invariant	D1	0.05	invariant
		D2	0.58	invariant	D2	0.07	invariant
		D3	-0.40	invariant	D3	0.00	invariant
		D4	-0.32	invariant	D4	-0.13	invariant
		D5	0.64	invariant	D5	0.47	invariant
		D7	0.71	invariant	D7	0.19	invariant
ACYPI001701	cytochrome P450 15A1	D0	0.85	invariant	D0	0.42	invariant
		D1	-0.22	invariant	D1	0.00	invariant
		D2	0.51	invariant	D2	**1.72**	**up***
		D3	0.16	invariant	D3	0.11	invariant
		D4	0.69	invariant	D4	0.42	invariant
		D5	**1.10**	**up**	D5	**1.45**	**up**
		D7	0.31	invariant	D7	-0.24	invariant
ACYPI010105	acetyl-coA transporter	D0	0.90	invariant	D0	0.21	invariant
		D1	**-1.69**	**down**	D1	**-1.79**	**down**
		D2	**-2.11**	**down**	D2	**-2.93**	**down***
		D3	0.85	invariant	D3	0.16	invariant
		D4	-0.41	invariant	D4	-0.52	invariant
		D5	**-1.75**	**down**	D5	**-2.02**	**down**
		D7	-0.43	invariant	D7	-0.76	invariant
