# Supplementary material for: Bacteriocyte Reprogramming to Cope With Nutritional Stress in a Phloem Sap Feeding Hemipteran, the Pea Aphid Acyrthosiphon pisum
Source: Front Physiol. 2018 Oct 25;9:1498. doi: 10.3389/fphys.2018.01498 (PMC6209921; doi:10.3389/fphys.2018.01498)
Supplement: Supplementary file 9 [file Data_Sheet_1.docx]

Supplementary Material

Bacteriocyte reprogramming to cope with nutritional stress in a phloem sap feeding hemipteran, the pea aphid *Acyrthosiphon pisum*

Stefano Colella^1,$,§,*^, Nicolas Parisot^1,$^, Pierre Simonet^1^, Karen Gaget^1^, Gabrielle Duport^1^, Patrice Baa-Puyoulet^1^, Yvan Rahbé^1,£^, Hubert Charles^1^, Gérard Febvay^1^, Patrick Callaerts^2^ and Federica Calevro^1,*^

^1^ Univ Lyon, INSA-Lyon, INRA, BF2I, UMR0203, F-69621, Villeurbanne, France.

^2^ KU Leuven, University of Leuven, Department of Human Genetics, Laboratory of Behavioral and Developmental Genetics, B-3000, Leuven, Belgium

*** Correspondence:**F Calevro, E-mail: [federica.calevro@insa-lyon.fr](mailto:federica.calevro@insa-lyon.fr); and S Colella, Email: [stefano.colella@inra.fr](mailto:stefano.colella@inra.fr). UMR0203 INRA/INSA de Lyon ‘Biologie Fonctionnelle, Insectes et Interactions’ (BF2I), 11 avenue Jean Capelle, F-69621, Villeurbanne, France. Phone: +33 472 43 79 88. Fax: +33 472 43 85 34.

**^$^ SC and NP should be considered joint first author.**

**^§^ Present address:** LSTM, Laboratoire des Symbioses Tropicales et Méditerranéennes, INRA, IRD, CIRAD, SupAgro, Univ Montpellier, Montpellier, France.

**^£^ Present address:** Univ Lyon CNRS, UMR5240 MAP, Microbiologie, Adaptation et Pathogénie, F-69622, France.

# Supplementary Figures

**Supplementary Figure 1.** **(A)** Principal component analysis (PCA) of all 66 available samples based on the expression profiles of the 24 011 transcripts analyzed in the microarray experiment. The percentage of variance explained by the first two principal components is shown in brackets. **(B)** Heatmap of expression profiles of the 24 011 transcripts analyzed in the microarray experiment and clustering analysis of all the 66 available samples. Hierarchical clustering of samples was performed using the euclidean distance with complete linkage method. The Gut−AP3_D3−1 sample showed an aberrant expression profile clearly distinct from the two others replicates of this time point in the gut tissue (labelled in black).

**Supplementary Figure 2.** Effects of a Tyr/Phe depleted diet (YFØ) on gene expression profiles across samples. Principal components analysis (PCA) of the 64 samples based on the expression profiles of the 24 011 transcripts analyzed in the microarray experiment. The percentage of variance explained by the first two principal components is shown in brackets.

**Supplementary Figure 3.** Correlation analysis between microarray and qRT-PCR data. The regression analysis using a linear model resulted in an adjusted R-squared of 0.8299 (F = 210.8; d.f. = 42; *P* < 2.2 × 10^-16^).
